# A Current Review of Machine Learning and Deep Learning Models in Oral Cancer Diagnosis: Recent Technologies, Open Challenges, and Future Research Directions

**DOI:** 10.3390/diagnostics13071353

**Published:** 2023-04-05

**Authors:** Shriniket Dixit, Anant Kumar, Kathiravan Srinivasan

**Affiliations:** 1 School of Computer Science and Engineering, Vellore Institute of Technology, Vellore 632014, India; 2 School of Bioscience and Technology, Vellore Institute of Technology, Vellore 632014, India

**Keywords:** artificial intelligence, computer-aided diagnostics, deep learning, machine learning, man–machine systems, oral cancer diagnosis

## Abstract

Cancer is a problematic global health issue with an extremely high fatality rate throughout the world. The application of various machine learning techniques that have appeared in the field of cancer diagnosis in recent years has provided meaningful insights into efficient and precise treatment decision-making. Due to rapid advancements in sequencing technologies, the detection of cancer based on gene expression data has improved over the years. Different types of cancer affect different parts of the body in different ways. Cancer that affects the mouth, lip, and upper throat is known as oral cancer, which is the sixth most prevalent form of cancer worldwide. India, Bangladesh, China, the United States, and Pakistan are the top five countries with the highest rates of oral cavity disease and lip cancer. The major causes of oral cancer are excessive use of tobacco and cigarette smoking. Many people’s lives can be saved if oral cancer (OC) can be detected early. Early identification and diagnosis could assist doctors in providing better patient care and effective treatment. OC screening may advance with the implementation of artificial intelligence (AI) techniques. AI can provide assistance to the oncology sector by accurately analyzing a large dataset from several imaging modalities. This review deals with the implementation of AI during the early stages of cancer for the proper detection and treatment of OC. Furthermore, performance evaluations of several DL and ML models have been carried out to show that the DL model can overcome the difficult challenges associated with early cancerous lesions in the mouth. For this review, we have followed the rules recommended for the extension of scoping reviews and meta-analyses (PRISMA-ScR). Examining the reference lists for the chosen articles helped us gather more details on the subject. Additionally, we discussed AI’s drawbacks and its potential use in research on oral cancer. There are methods for reducing risk factors, such as reducing the use of tobacco and alcohol, as well as immunization against HPV infection to avoid oral cancer, or to lessen the burden of the disease. Additionally, officious methods for preventing oral diseases include training programs for doctors and patients as well as facilitating early diagnosis via screening high-risk populations for the disease.

## 1. Introduction

Oral cancer is a type of head and neck cancer that affects the mouth, tongue, lips, and throat. According to the World Health Organization (WHO), oral cancer is the sixth most common cancer worldwide, with an estimated 300,000 new cases diagnosed each year [1]. The incidence of oral cancer varies widely between different regions of the world, with the highest rates being reported in South and Southeast Asia. The American Cancer Society (ACS) estimates that in the United States, approximately 54,540 people will be diagnosed with oral or oropharyngeal cancer this year. The death rate for oral cancer is relatively high, with approximately 11,580 deaths expected to occur in 2023. Risk factors for oral cancer include tobacco use (smoking and smokeless tobacco), excessive alcohol consumption, human papillomavirus (HPV) infection, and a diet low in fruits and vegetables. Men are more likely to develop oral cancer than women, and the risk increases with age. Early detection and treatment of oral cancer are important for improving outcomes. The five-year relative survival rate for oral cancer is approximately 90% when the cancer is detected early and treated appropriately [2]. However, when the cancer is diagnosed at a later stage, the five-year survival rate drops significantly. In India, the survival rate for oral cancer (OC) has improved due to early diagnosis and treatment [3]. The insides of the cheeks, lips, gum line, and the space at the back of the adult teeth are all potential growth sites for OC. A blister or ulcer that does not heal and may cause pain or bleeding is the most common sign of malignancy. Some of the common symptoms of OC include white or red sores; non-healing sores on the lips, gum line, or tongue; a lump inside the mouth; weak teeth; difficulty in chewing or swallowing; trouble speaking; discomfort in the jaw; and a persistent sore throat. One of the earliest signs of OC is the development of leukoplakia [4]. Oral cancer is a significant health concern in India, with a high incidence rate and a relatively high mortality rate compared with many other countries. According to the Indian Council of Medical Research (ICMR), oral cancer is the most common cancer among men and the second most common cancer among women in India. According to GLOBOCAN 2020, oral cancer accounted for about 10.3% of total cancers reported in 2020. It led to 75,290 deaths and ranked second. The five-year prevalence is 300,413. In general, more men suffer and die from oral cancer than women [5]. Tobacco use including smoking cigarettes, pipes, and cigars, and using smokeless tobacco products such as chew, and snuff is one of the primary risk factors for oral cancer. The use of tobacco products is a leading cause of cancer globally, including oral cancer [6].

Another important risk factor for oral cancer is excessive alcohol consumption, which can independently increase the risk of developing oral cancer; however, when it is combined with tobacco use, the risk increases dramatically. Infection with human papillomavirus (HPV) is also a risk factor for oral cancer, particularly oropharyngeal cancer (a type of cancer that affects the back of the throat, including the base of the tongue and tonsils). Furthermore, a diet low in fruits and vegetables, exposure to wood smoke and other environmental pollutants, and poor oral hygiene are also considered risk factors for oral cancer [7]. It is worth noting that the relationship between cancer in general and oral cancer is complex and multifactorial, and not all oral cancer cases can be attributed to known risk factors. Early detection and treatment of oral cancer, as well as efforts to reduce risk factors such as tobacco and alcohol use, are crucial for improving outcomes for patients with oral cancer. In India, oral cancer is often diagnosed at a late stage, leading to a relatively low survival rate. The five-year survival rate for oral cancer in India is estimated to be around 20%. The most commonly used method for diagnosing oral cancer is a hematoxylin–eosin histological examination, which is combined with taking a standard medical history and carrying out a clinical assessment [3]. Although the detection of oral cancer in its early stages is crucial, most patients experience poor prognoses due to late detection. The selection of treatment, which is primarily based on the disease’s clinical presentation, is insufficient as the clinical manifestation of oral cancer is not a reliable indicator of the disease’s status, progression, or dysplastic degree. There are numerous factors linked to the disease, and the success of treatment is likewise uncertain [8]. Scientists have developed various classifications to distinguish benign from malignant oral lesions. The Apriori Algorithm has shown that all the created rules have a maximum level of confidence, making them highly useful for the early detection and prevention of oral cancer [9].

Various imaging techniques can capture images of oral lesions in different forms. A dual-mode image processor that integrates white light imaging and autofluorescence has been proposed in the literature as a method for detecting oral cancer [10]. Oral lesions that are present in the later stages of the disease have a negative impact on survival rates, with over two-thirds of oral lesions being detected at a later stage [11]. The cost of lesion care is high, particularly in the later stages [12]. Late diagnosis of oral lesions is a significant concern for medical professionals [13]. To improve the early detection of oral cancer and reduce the impact of late diagnosis, it is important to develop an automated system for the detection of OC with minimal human interaction. Machine learning (ML) has been demonstrated to be useful in increasing classification accuracy in automated systems. In particular, deep learning (DL) has been shown to reduce the need for human involvement in the analysis of large datasets [14]. AI has the potential to address current challenges in the identification and prognosis prediction of oral cancer, by reducing the workload, complexity, and fatigue for doctors during the diagnostic process. It is a technical advancement that has garnered significant interest from scientists worldwide as it simulates human cognitive abilities. Its application in dentistry is a relatively recent development, but the results have been promising. Aubreville et al. [15] used DL algorithms to analyze confocal laser endomicroscopic images and detect oral squamous cell cancer. The authors in [16] also utilized a deep architecture to diagnose dysplasia in microscopic images.

AI has the potential to help solve some of the challenges associated with the diagnosis of oral cancer by improving the accuracy and efficiency of diagnostic tools. In summary, AI-based diagnostic tools have the potential to address several challenges associated with the diagnosis of oral cancer, including the lack of symptoms in the early stages, difficulty in visualizing the oral cavity, limited resources, lack of awareness, limited diagnostic tools, and difficulty in distinguishing benign from malignant lesions. These tools can improve the accuracy and efficiency of oral cancer diagnosis by analyzing medical images, identifying biomarkers, predicting patient outcomes, and differentiating benign from malignant lesions. They can also be integrated into existing healthcare systems and can be used to analyze images and data remotely, to overcome limitations associated with limited resources.

### 1.1. Objectives

In this study, various AI, ML, and DL classifiers for the early detection of oral cancer have been presented and discussed. Previous research has demonstrated the potential of ML models in oral cancer prediction and diagnostic analysis. Deep hybrid learning approaches are also being utilized to enhance the understanding of these models and externally verify them for generalization before their incorporation into routine therapeutic operations. While previous studies have primarily focused on the ML and DL aspects of oral cancer diagnosis, they have been superficial in their methodology. This survey, however, has been compiled in a comprehensive manner to be useful for early researchers interested in this domain. To the best of our knowledge, there is no single paper that has discussed the combination of ML and DL extensively alongside recent trends in technologies related to oral cancer diagnosis. This review is focused on the application of ML and DL techniques in the diagnosis of oral cancer. It provides a comprehensive overview of the existing data collection techniques, their limitations, and the various ML and DL techniques that can be used in this field. Unlike other systemic reviews that cover similar topics [17,18,19], this review has specifically addressed the different types of oral cancer and provided a detailed analysis of the limitations of current ML and DL models. Additionally, this review has identified open challenges in AI-based diagnosis technologies and proposed future research directions. This comprehensive study aimed to provide an in-depth analysis of ML and DL models for automated oral cancer diagnosis and to further advocate these models as possible modern technologies for medical decision support systems.

### 1.2. Current Insights into Oral Cancer Diagnostics

The diagnosis of oral cancer poses several challenges that can impede the early detection of the disease. As mentioned above, these challenges include the absence of symptoms during the early stages of the disease, difficulties in visualizing the oral cavity, limited resources, inadequate awareness, a lack of appropriate diagnostic tools, and difficulties in distinguishing benign from malignant lesions. Despite the importance of early diagnosis for optimal patient outcomes, the diagnosis of oral cancer remains a challenge for healthcare professionals due to the aforementioned obstacles [20]. There are several challenges associated with using ML for the diagnosis of oral cancer. These include the limited availability of high-quality and diverse training data, issues with data quality, the tendency for models to overfit, and difficulties in interpreting the models. These challenges can negatively impact the performance and reliability of the models. In addition to these challenges, the development and implementation of ML models for the diagnosis of oral cancer are also hindered by a lack of consensus on the optimal training algorithms [18,19,20,21,22,23,24,25]. This makes it difficult to compare the accuracy of different models across studies. One solution to this problem is the creation and development of large patient datasets for prognostic analysis. However, this requires dedicated infrastructure for data storage and the consideration of healthcare data privacy. Additionally, the support of political governments and the scientific community, and individual awareness are also necessary. To advance research in this field, a multidisciplinary team of researchers with expertise in both ML and biological research is required. Table 1 presents the list of abbreviations used in this manuscript along with their full form.

### 1.3. Contribution of This Review

In this work, we ascertained how ML and DL are used to identify oral cancer. We included several regional oral cancers.

The following is a simple summary of our contributions:

- We carried out a review of recent methods for detecting the early signs of oral cancer, including extreme learning machines, DBN, the deep generative model, and others. Furthermore, more traditional methods from the field of artificial intelligence, including random forest, ANN, DNN, KNN, and others were also included.
- We used an extensive tabular style to describe the studies on the use of ML and DL in OC. The summary includes information regarding the model, significant contributions, and model constraints.
- This review specifically addressed current issues and potential solutions for diagnosing and treating oral cancer.
- Table 2 compares the current review with earlier surveys or other review articles of a similar nature.

### 1.4. Survey Methodology

#### 1.4.1. Search Strategy and the Literature Sources

**Figure 1:**
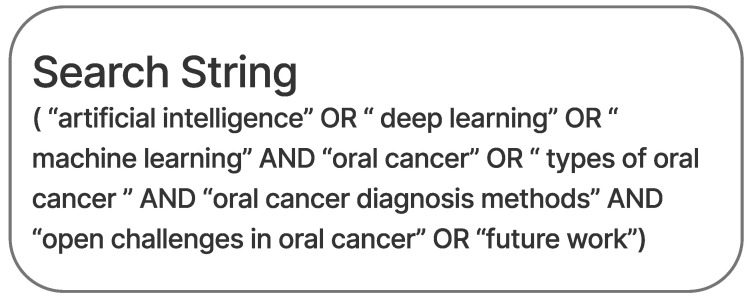
Inquiries made using key terms in databases.

This study has presented many research findings and studies involving the used of ML and DL methods to diagnose oral cancer that have been published in multiple online databases (Science direct, PubMed Central, IEEE, etc.). The keywords used for the database queries are shown in Figure 1. It was observed that the number of papers discussing OC diagnosis utilizing DL techniques has been steadily increasing in recent years. Moreover, this evaluation analyzed the work of other academics and suggested novel research directions.

#### 1.4.2. Inclusion Criteria

Only English-language publications were taken into consideration, and the selection was based on the articles’ eligibility as well as the novelty of the review subject. Moreover, case reports, case studies, opinions, and letters to the editor were not included in this evaluation.

#### 1.4.3. Elimination Criteria

Abstract screening was used for the first phase of exclusion, while data extraction and full texts were used for the second round. The articles were then rejected because they were irrelevant, were written in a language other than English, or were poorly written.

#### 1.4.4. Results

**Figure 2:**
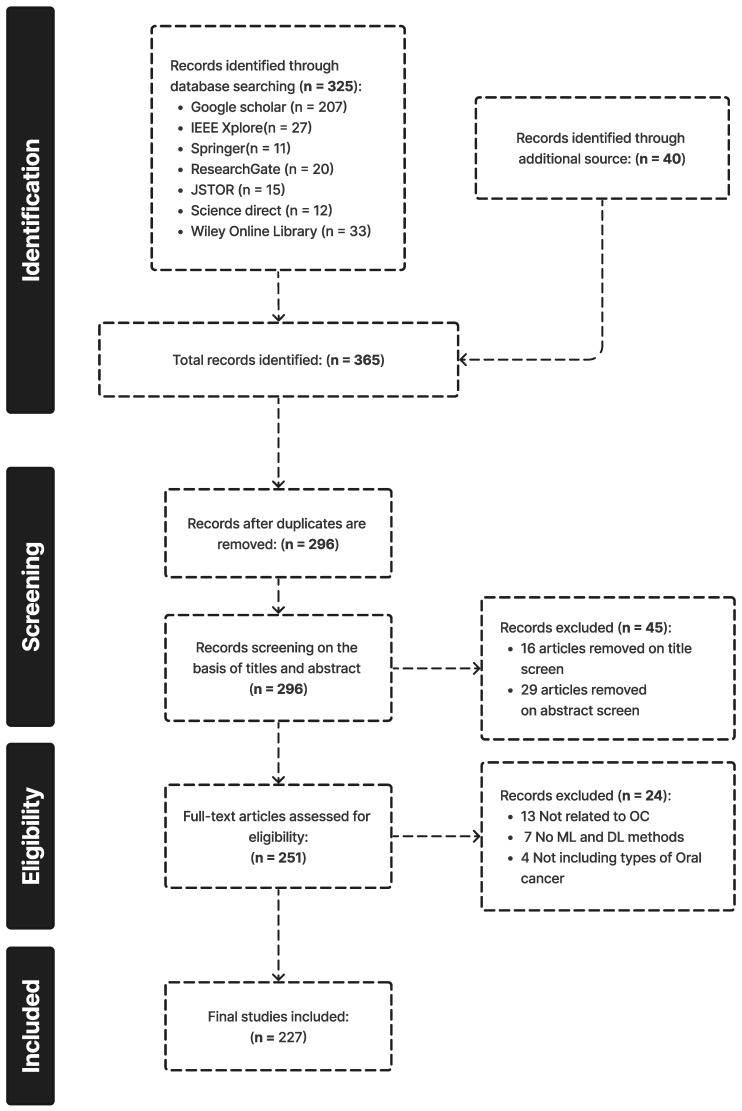
Articles were identified using the PRISMA method.

**Figure 3:**
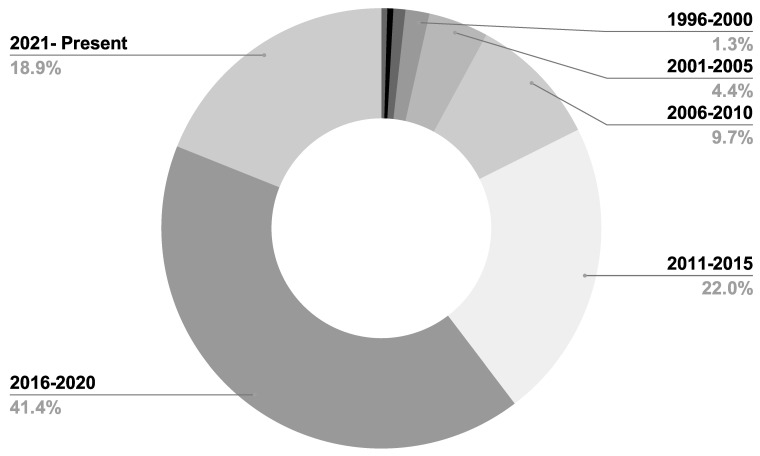
Number of papers per year, used in the review.

**Figure 4:**
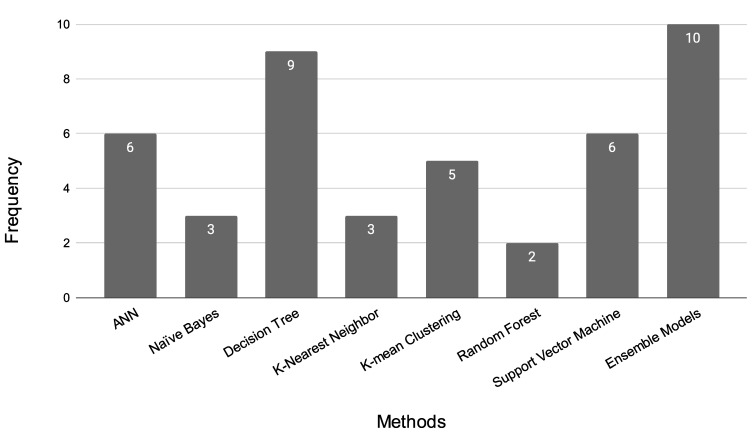
ML models vs. frequency of papers used in this work.

**Figure 5:**
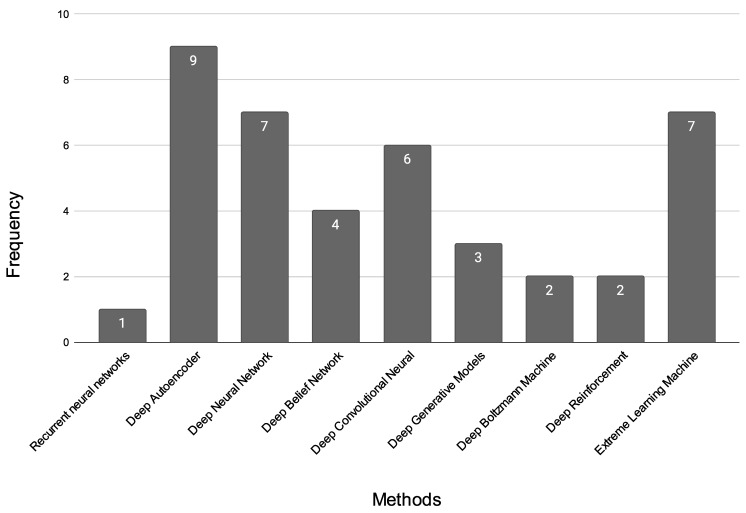
DL models vs. frequency of papers used in this work.

**Figure 6:**
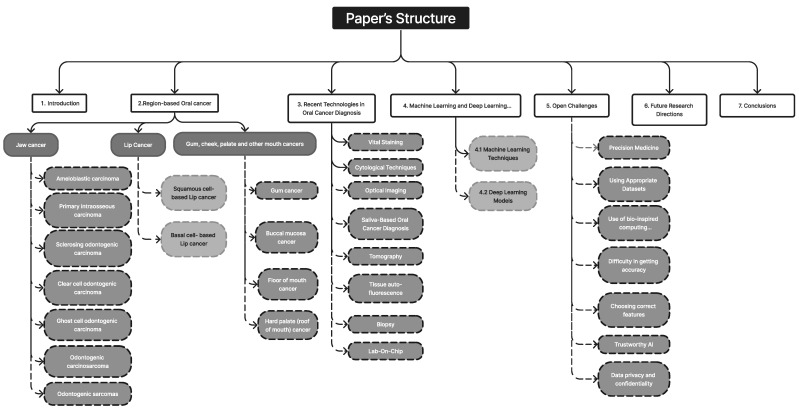
Structure of this article.

A total of 296 unique publications were gathered from Google Scholar, IEEE Xplore, Springer, and other literary sources; following title and abstract screening, 45 articles were disqualified. After a full-text analysis of the remaining 251 articles, an additional 24 papers were discarded, leaving 227 articles for consideration in the final evaluation [1,2,3,4,5,6,7,8,9,10,11,12,13,14,15,16,17,18,19,20,21,22,23,24,25,26,27,28,29,30,31,32,33,34,35,36,37,38,39,40,41,42,43,44,45,46,47,48,49,50,51,52,53,54,55,56,57,58,59,60,61,62,63,64,65,66,67,68,69,70,71,72,73,74,75,76,77,78,79,80,81,82,83,84,85,86,87,88,89,90,91,92,93,94,95,96,97,98,99,100,101,102,103,104,105,106,107,108,109,110,111,112,113,114,115,116,117,118,119,120,121,122,123,124,125,126,127,128,129,130,131,132,133,134,135,136,137,138,139,140,141,142,143,144,145,146,147,148,149,150,151,152,153,154,155,156,157,158,159,160,161,162,163,164,165,166,167,168,169,170,171,172,173,174,175,176,177,178,179,180,181,182,183,184,185,186,187,188,189,190,191,192,193,194,195,196,197,198,199,200,201,202,203,204,205,206,207,208,209,210,211,212,213,214,215,216,217,218,219,220,221,222,223,224,225,226,227]. The outcomes are displayed in Figure 2. Figure 3, Figure 4 and Figure 5 show the overall analysis of the 227 studies included. The overall framework of this review is shown in Figure 6.

## 2. Region-Based Oral Cancer

### 2.1. Lip Cancer

Lip cancer, also known as squamous cell carcinoma (SCC) of the lip, is a prevalent form of oral malignancy that occurs in the oral–maxillary region. In recent years, there has been a significant increase in the incidence of lip cancer. The authors in [28] examined the epidemiological data and risk factors for lip cancer among patients who visited their departments between 2000 and 2010. Out of 540 cases, most patients (84.8%) were men and women over 45 years old. The research revealed that individuals with SCC displayed the typical clinical and epidemiological features identified in prior investigations. These findings can be used to inform the development of methods for health promotion and to raise awareness of the risk factors associated with lip cancer.

#### 2.1.1. Squamous-Cell-Based Lip Cancer

Squamous-cell-based lip cancer typically presents as well-differentiated lesions that spread later in their growth, with a tendency for metastasis to the submandibular or submental nodes on the opposite side [34]. The lower lip (85–95%) is the most affected area, with the upper lip (2–7%) and lip sulcus (1–4%) being the next most frequently affected areas. Lip cancer can resemble an unhealing mouth sore, which may appear red on individuals with light skin or deep brown or grey on individuals with darker complexions [35]. Surgery to remove the malignancy is typically the first step in treatment, with more invasive treatments being used for the advanced stages [36]. However, these treatments can result in higher morbidity and treatment costs, as well as a significant reduction in the patient’s quality of life. In the later stages of malignancy, the prognosis for oral cancer (OC) deteriorates. According to studies, SCCs make up approximately 95% of non-melanoma skin malignancies and commonly occur on the lower lip [33]. In countries with low to moderate incomes, particularly in Southeast and South Asia, oral cancer accounts for two-thirds of cases [1]. Basal cell carcinomas, which are less common, tend to impact the upper lip, but typically do not involve lymph node metastases [37].

Most cases of OSCC arise from oral potentially malignant disorders (OPMDs) in the oral cavity, which have a 1% chance of transforming into malignant lesions such as erythroleukoplakia, oral lichen planus, and leukoplakia [30]. Early detection of OSCC is crucial for a successful treatment plan, improved prognosis, and low rates of death and morbidity [38]. The prognosis for advanced stages of oral cancer is poor, with a 50% typical therapeutic efficacy [7,39]. Histological analysis of tissue samples is the primary method for diagnosing OSCC, but this approach is time-consuming and prone to error [40,41]. Therefore, it is important to provide pathologists with effective diagnostic tools to aid in the assessment and treatment of OSCC. The use of DL technology for the detection and classification of OSCC has been shown to have great potential for improving treatment efficiency and accuracy [42].

#### 2.1.2. Basal-Cell-Based Lip Cancer

Basal-cell-based lip cancer is characterized by multiple basal cell carcinomas and is also associated with malformations affecting various systems of the body, including the heart, genitourinary system, central nervous system, and skeletal system, as well as specific physical features such as frontal bossing, a large nasal bridge, macrocephaly, and a projecting jaw [43]. BCC is a locally invasive tumor that arises from stem cells in the interfollicular epidermis and hair follicles and presents as plaques or nodules on the skin [44]. It is important to detect and treat BCCs early, through routine skin cancer screening and sun protection, and to minimize ionizing radiation exposure [45]. Orbital invasion, which is associated with less than 5% of periocular BCCs, can occur with advanced BCCs or tumors that continue to grow perineurally [46]. Factors associated with a higher likelihood of orbital invasion include a primary tumor near the medial canthal, perineural infiltration, an infiltrative or sclerosing BCC, and multiple recurrences [47].

### 2.2. Jaw Cancer

Jaw cancer can be caused by the spread of other head and neck cancers or by a rare type of bone cancer called osteosarcoma. Jaw-related bone tumors make up approximately 1% of all head and neck malignancies. The five-year survival rate is typically between 25% and 40%, with mandibular tumors having a better prognosis than maxillary tumors [48]. Treatment options include surgery, radiation, and/or chemotherapy, with surgery being the first-line treatment. The risk of recurrence is higher in patients who are diagnosed at later stages, emphasizing the importance of early detection for a positive outcome [49].

#### 2.2.1. Ameloblastic Carcinoma

Ameloblastic carcinoma (AC) is a rare and malignant odontogenic tumor that can be difficult to distinguish from the benign tumor, ameloblastoma. Conversion from ameloblastoma to AC is believed to occur over a long period and may involve multiple stages of carcinogenesis [50,51]. In recent years, there have been an increasing number of reported cases of AC, but this may be due to improved recognition and diagnosis rather than a true increase in incidence [52]. Repeated surgical treatment for odontogenic tumors in resource-limited settings can also lead to secondary cases of AC. It may present as an ulcerated, massive tissue mass with mobile teeth and bone resorption, or as a benign-appearing cystic lesion, making it difficult to diagnose. Dental professionals need to be aware of and recognize the distinguishing characteristics of AC [51,53,54]. It has distinct histological characteristics such as epithelial tumor islands and nests encircled by a layer of stellate basaloid cells during the early stages of malignancy or dedifferentiation, and the signs of malignancy include perineural invasion, cellular focal necrosis, mitoses, pleomorphism, and nuclear hyperchromatism.

The exact mechanism of how AC develops from ameloblastoma is not well understood [55]. Younger individuals have a higher survival rate, and primary AC has a better prognosis than secondary AC [56]. Adjuvant radiation or chemotherapy may not be more effective than standard postoperative care, but they should be considered for locally advanced or metastatic cases [57]. Surgical resection is the most effective treatment for AC, as it has a lower recurrence rate compared to alternative conservative therapies [55]. Early diagnosis and aggressive total excision of the tumor are recommended to increase survival rate. FDG PET/CT testing should be carried out during the diagnostic process to detect potential metastatic disease [58].

#### 2.2.2. Primary Intraosseous Carcinoma

PIOC was first described as a core epidermoid cancer of the jaw in 1913 by Loos, and was later referred to as primary intra-alveolar epidermoid cancer by the World Health Organization (WHO) in 1971 [59]. In addition to radiation and chemotherapy, broad local excision is the mainstay of treatment for PIOC. In a previous study, at the initial examination, there was no evidence of regional lymph node enlargement in either patient being studied. However, 1 week after the tumors were treated, ipsilateral lymphadenopathy was noticed. The patients were treated with a hemimandibulectomy in these instances, in addition to a radical neck dissection [60]. PIOC has distinct histological characteristics, including features that indicate that the tumor originates from the odontogenic epithelium [61]. The condition is more common in males and typically affects adults over the age of 50 years; however, it is rare in pediatric and adolescent populations [61]. PIOC is sometimes mistaken for an odontogenic cyst since it occasionally displays well-defined boundaries in imaging tests [62].

#### 2.2.3. Sclerosing Odontogenic Carcinoma

The most recent edition of the WHO classification of neck and head malignancies now includes sclerosing odontogenic carcinoma (SOC) which is a rare type of primary intraosseous cancer of the jaw [63]. Despite being recognized by the WHO as a unique entity, there have only been a small number of documented cases of this tumor in the literature [64,65,66,67,68,69]. The histomorphology of SOC includes mixed epithelial and mesenchymal components, resembling other odontogenic neoplasms, and presents a diagnostic problem due to the histologic overlap [63,67,69,70]. It should be treated as an excluding diagnosis and the treatment for this type of tumor is still challenging due to its rarity and locally invasive nature.

#### 2.2.4. Clear Cell Odontogenic Carcinoma

Clear cell odontogenic carcinoma (CCOC) originates in the dental lamina, and it was first identified as such in 1985 [71,72,73]. It may be difficult to distinguish from other types of cancer due to its similarities in histology and immunology. It is more commonly found in the mandibular and maxillary bones and more frequently found in women [71]. The treatment options include surgical resection, but recurrence and metastasis rates are high [74,75,76]. In a previous study, the metastatic lesion resembled the initial biopsy in appearance, and the diagnosis was made after comparing histological characteristics and immunohistochemistry, and ruling out a second main tumor through clinical assessment and radiology [77]. There is little evidence to support the use of chemotherapy as a curative treatment; hence, it should only be used to relieve symptoms. For CCOC, moderate radiotherapy is also beneficial [78]. The primary method of treatment is surgical resection with a wide margin. Other options include curettage or surgical excision, laparotomy, post-operative radiation, and chemotherapy [79]. The WHO reclassified CCOC as a “malignant carcinoma” of odontogenic origin in 2005 due to its aggressive and locally destructive behavior, which can sometimes metastasize [80]. The CCOC’s histopathologic characteristics clearly indicate that this entity has an inductive capacity on the ectomesenchyme as well as a pathogenetic interaction with the ameloblastic epithelium [81]. Differential diagnoses include diseases of the odontogenic epithelium, salivary gland disorders, and distant organ metastases. However, recent research has suggested that CCOC may represent a low-grade sarcoma and the bone equivalent of hyalinizing clear cell carcinoma associated with the salivary gland [82,83].

#### 2.2.5. Ghost Cell Odontogenic Carcinoma (GCOC)

GCOC, or ghost cell odontogenic carcinoma, is an aggressive type of malignant odontogenic tumor that develops from odontogenic ghost cells [84]. It is characterized by ghost cells, malignant cellular characteristics, dental deposits, and areas of invasion and necrosis. It can develop from benign odontogenic neoplasms such as calcifying odontogenic cysts or dentinogenic ghost cell tumors [85,86]. The diagnosis of GCOC can be difficult due to its similarities with other odontogenic ghost cell lesions, and its nonspecific radiologic and clinical features [86]. Its treatment usually involves a combination of aggressive surgical strategies and a multimodal treatment plan.

#### 2.2.6. Odontogenic Carcinosarcoma

Odontogenic carcinosarcoma (OCS) is a very rare form of jaw cancer that is made up of both epithelial and mesenchymal components [87,88]. It is similar in structure to ameloblastic fibroma (AF) and can appear as a new lesion or develop from an existing odontogenic lesion [87,89]. The histological features of OCS include malignant epithelial cells present in hypercellular mesenchymal tissue, and hyperchromatic and pleomorphic cells. OCS can be mistaken for other types of odontogenic tumors and has a high rate of recurrence and metastases [89]. The WHO initially classified OCS as a unique entity in 1992, but later withdrew it in the third edition of its classification in 2005 due to difficulty in distinguishing it from other tumors; it was later reinstated as a unique entity in the most recent classification by WHO in 2017 [90,91]. The pathophysiology, incidence, and outcome of OCS are not well understood, and more research is needed to improve diagnosis and treatment.

#### 2.2.7. Odontogenic Sarcomas

Ameloblastic fibrosarcoma (AF), also known as odontogenic sarcoma, is a malignant odontogenic tumor. It typically consists of a normal epithelial component and a malignant mesenchymal component [92]. A case report has documented a rare instance of AFS that developed from an AF [93]. Due to the rarity of metastasis, AFS is considered a low-grade tumor, but it still requires aggressive surgical treatment and postoperative care including chemotherapy and radiation therapy [94]. It is important to treat AF promptly to prevent its transformation into AFS, and to monitor patients with both AF and AFS over a prolonged period.

### 2.3. Gum, Cheek, Palate, and Other Mouth Cancers

#### 2.3.1. Gum Cancer

Gum cancer, also known as oral malignancy, affects the gum tissue and makes up 6% of oral cancers. Symptoms include lumps, bleeding, and white or ulcerative lesions on the gums. Adenoid cystic carcinoma is the most common type, making up 15% of cases, followed by mucoepidermoid carcinoma (54%) and low-grade adenocarcinoma (17%) [95]. However, attrition from recurrent primary cancers in long-term survivors has hindered further advancements in survival. To improve survival rates, and increased knowledge and methods for early detection, as well as provide education concerning lifestyle-related risk factors are necessary [96].

#### 2.3.2. Buccal Mucosa (Inner Cheek) Cancer

The inner lining of the cheeks, known as the buccal mucosa, is an area that can develop cancer. Buccal mucosal cancer is a rare and dangerous form of oral cancer that makes up a small percentage of patients diagnosed with mouth cancer [97]. There is a correlation between local relapse and margin condition for both small and large buccal cancers that are treated only with surgery; however, adjuvant therapy can improve local control in early stage buccal cancers [98]. In young people, SCC of the buccal mucosa is preventable and easy to identify, although it often presents in the advanced stages due to socioeconomic factors.

#### 2.3.3. Floor of the Mouth Cancer

Treatment options for this type of cancer include local resection with radiation therapy and neck incision, local resection with radiation therapy, and composite resection with radiation therapy. However, a study found that none of these treatment options offered a significant survival benefit for patients with FOM cancer [99]. Cancers of the FOM can be challenging to treat due to the tendency for metastasis to bilateral cervical lymph nodes and the difficulty in reaching and resecting these tumors [100,101]. Additionally, FOM cancer is often combined with cancer of the oral tongue, and independent examination of these two types of cancer is preferred for treatment [102,103,104].

#### 2.3.4. Hard Palate (Roof of the Mouth) Cancer

**Figure 7:**
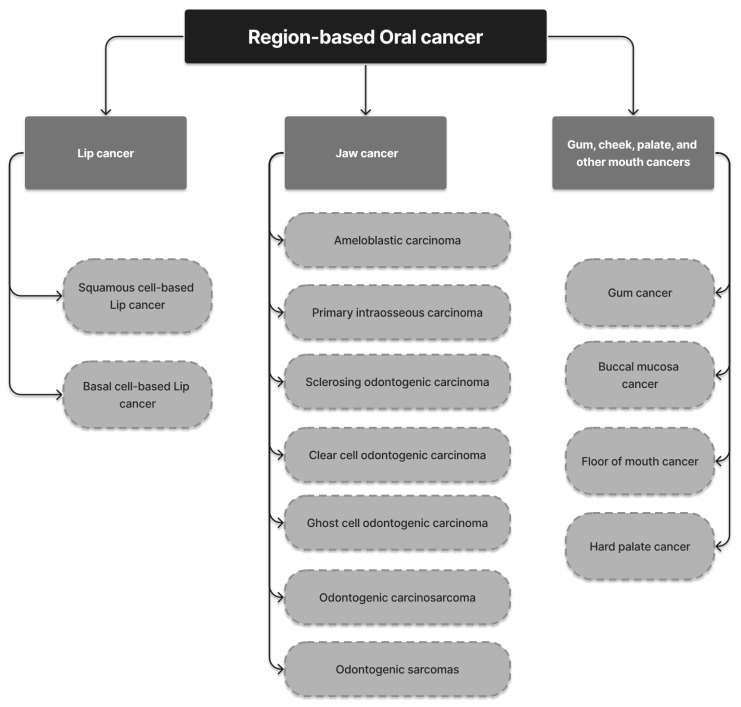
Classification of OC based on the origin location of lesion.

Hard palate cancer, a type of cancer that affects the bony portion of the roof of the mouth, occurs when cells in the area become uncontrollably proliferated and form tumors or lesions [105]. Risk factors for hard palate cancer include the use of tobacco products and excessive alcohol consumption. Early detection of hard palate cancer increases the chances of successful treatment. Surgery by a specialist in neck and head cancer is commonly used to treat the disease, to remove the cancer while preserving the appearance and function of the mouth. The treatment plan is determined by the depth and extent of the cancer and may include a procedure called a maxillectomy. In some cases, radiation therapy alone may be the only option for treatment [106]. Figure 7 illustrates the various categories of oral cancer grouped according to the origin location within the body.

## 3. Recent Technologies in Oral Cancer Diagnosis

**Figure 8:**
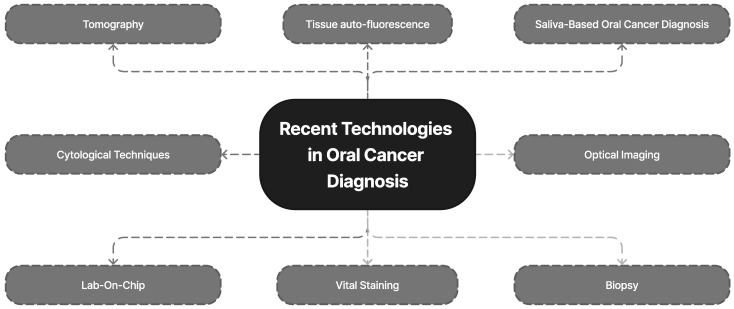
Technologies related to diagnosis of OC.

AI technology has been used to improve the early detection of oral cancer (OC). This is carried out by simulating human thinking and behavior using machines. DL, which is based on patterns found in the neocortex of the human brain, utilizes layers of artificial neural networks (ANNs) to improve picture interpretation [107,108]. The STARD 2015 report on diagnostic accuracy studies has also contributed to increased thoroughness and transparency in reporting [109]. The number of layers present in a DL model has a significant impact on its performance and complexity. Computers trained with DL can quickly execute a variety of algorithms and are being used for automated medical diagnostics. Figure 8 illustrates the modern methods for detecting oral cancer.

### 3.1. Visual Staining

Another technique for detecting malignancy is visual tissue staining [110]. This method is useful for developing nations such as India because of its simplicity and affordability. Toluidine blue combined with iodine assists in identifying inflammatory lesions, which is a crucial visual staining approach for the pre-therapeutic evaluation of OC [111].

### 3.2. Cytological Techniques

Oral cytology has once again become a major area of study throughout the last few decades. However, examination of the surface epithelial cells of the oropharynx and the oral cavity by conventional exfoliative cytology has thus far proven ineffective. This is contrary to the results associated with applying this technique to a sample of cells that belong to the uterine cervix. The mouth cavity’s form prevents a thorough examination of the mucous membrane. Conventional exfoliative cytology has been unable to gain access to the oral cavity’s deeper cell layers whist still remaining minimally invasive [112].

### 3.3. Optical Imaging

OPMD and OC detection methods based on light have been utilized in clinical settings for several years. To locate oral mucosal lesions, an intraoral detector that uses fluorescent or chemiluminescent light is used. To evaluate the health of the oral epithelium, the color of the light it reflects is employed [113].

### 3.4. Saliva-Based Oral Cancer Diagnosis

Saliva offers many advantages over serum as a diagnostic tool, including the ability to screen large populations in a feasible and affordable way. Saliva can be used to assess proteomic or genomic targets such as proteins, cytokeratins, telomerase, endothelin, metalloproteinases, growth factors, cytokines, DNAs, and mRNAs transcripts, as well as to evaluate certain salivary macromolecules [114,115]. A device can be used to remove all salivary lymphocytic cells from the sample using antibodies (attached to magnetic beads) that are specific to dysplasia and malignancy [116]. These antibodies can then be used to detect and enrich precancer and cancer cells. A combination of many salivary biomarkers, including mRNAs and proteins such as IL-8 and thioredoxin, can identify OC with high specificity and accuracy [117].

### 3.5. Tomography

To create a cross-sectional structural representation of tissue, OCT captures subsurface reflections. Gold nanoparticles with surface plasmon resonance can be used to improve the contrast in these photographs. It was discovered that this multimodal administration of antibody-conjugated polyethylene-glycol-linked gold nanoparticles improved the contrast of in-vivo OCT pictures of oral dysplasia present in a hamster model [118]. Many pilot studies have been conducted to explore the potential of OCT images in oncology [119,120,121]. These images can also be used in DL models to automate the diagnosis process and make it more reliable.

### 3.6. Tissue Auto-Fluorescence

The oral mucosa changes biochemically and anatomically as OC progresses [114]. The interplay of light with these altered parameters during tissue auto-fluorescence provides complex information about whether the lesion is precancerous or cancerous [113]. Fluorescence results from strong blue light (400–430 nm) being absorbed by the normal mouth mucosa and re-emitted at a longer wavelength. The levels of metabolism and the collagen in the stroma of NADH and FAD are both responsible for the fluorescence signal generated. These complex features can be extracted by DL models and help in the diagnosis process.

### 3.7. Biopsy

Biopsy is the only approach to detect oral or oropharyngeal cancer [122]. For a specific histological diagnosis, the tissue must be handled carefully during a biopsy. A faulty biopsy may result from improper sample management, necessitating repeated surgery. Depending on the specific necessity, incisional biopsy and exfoliative cytology are the two main types of biopsies that are carried out. Even biopsy images can be analyzed using ML or DL models, which can further help with quick decision making and accurate diagnosis.

### 3.8. Lab-On-Chip

A special microelectromechanical device called a LOC can be utilized to find ribonucleic acid (RNA) and protein biomarkers. It makes use of microfluidic engineering techniques that automate the separation and preparation of specimens such as saliva, urine, blood, serum, signal detection, and signal transduction on a single chip [123,124] LOC platforms have been used for a variety of immunoassays, biochemical processes, and molecular diagnostics, including nucleic cell sorting, protein assays, acid assays, etc. [123]. LOC is also associated with a reasonably low cost, and improves reproducibility and consistency [124]. OC diagnoses will be significantly impacted by LOC. In a previous study, a mobile tablet microscope was created as a tool for screening OC. This mobile microscope was utilized to scan a slide sample and take high-resolution pictures of stained brush biopsy samples. It was coupled with an iPad Mini that had Bluetooth-controlled motors, LED lighting, and collection optics. This technology may increase screening efficiency in remote areas and healthcare settings without specialists since the results showed agreement in image evaluation, cytology, and histology compared with those of a distant pathologist [125,126].

## 4. Machine Learning and Deep Learning Models for Oral Cancer Diagnosis

### 4.1. Machine Learning Techniques

#### 4.1.1. Artificial Neural Network

The contribution of an ANN is essential for the development of risk prediction in OC. Due to an ANN’s advantages in data processing, it has been widely applied in various fields. It is clear that using neural networks for screening reduces the time required of experienced doctors and aids in the identification of high-risk categories. In [127], an ANN and oral precancerous lesions were integrated together and suitable theoretical formulations were employed to explain the results. Its output (90% accuracy) showed that the strategy was more reliable than traditional prediction methods. This algorithm can diagnose OC lesions in high-risk populations. Singh et al. [128] carried out the categorization using first-order and second-order statistical data as well as textural features. An oral precancerous lesions risk diagnostic model was created in this study using an ANN. In [129], an automated diagnosis system was constructed using DNNs and ML models. Naive Bayes, KNN, SVM, ANN, and CNN classification techniques were built for the automated classification and detection of OCs, utilizing the initial data that were acquired in this work. The conventional VGG-16 network served as the basis for the creation of a new CNN network with 43 deep layers. The ANN performed better than other techniques due to its precision of 99.4%. The findings of the authors in [130] have shown that by using information regarding a person’s risk factors, clinicopathological data, and systemic health issues, an ANN can forecast an individual’s risk of developing OC. The findings showed that the ANN model could excel in predicting the likelihood of malignancy and enhance the positive predictive value (PPV). This may provide medical professionals with a straightforward tool to assist with predicting a person’s OC risk depending on their understanding of their clinicopathological statistics, systemic health problems, and risk factors.

To create a reliable predictive model that is able to enhance OC diagnosis and screening, additional studies with larger cohorts are required. A previous study has used features from standard techniques (GLCM, FCH, LBP, and DWT) combined with hybrid features extracted from ResNet-18 and AlexNet models to accurately diagnose histopathology images of OC. The hybrid features were then fed into an ANN network for classification. It is important to consider that this strategy trains the required data set quickly. In total, 73 patients qualified for this experiment. A total of 52 instances (69.86%) were malignant, compared to 22 benign cases (30.13%). With an average age of 63.09 years, there were 37 female participants and 36 male participants. Based on the examination of 10-fold cross-validation, this investigation showed that ANN’s typical sensitivity and specificity for OC prediction was 78.95%. A study was carried out by the authors in [131] used an ANN and cytology slide digitalization as the technique for early detection of OC. AI’s efficacy was compared to that of conventional cytology and histology. Based on the risk categorization model, 11,981 prepossessed pictures were used in this study. The ANN-based model increased the accuracy of malignant detection by 93% and had an accuracy of 73% for potentially malignant lesions. Tseng et al. [132] distinguished between the symptoms displayed by OC patients who died and those who survived. For 674 OC patients, the effectiveness of an ANN, DTs, and conventional logistic regression were examined. Prognostic variables included in the study included survival rates, fatalities, cancer incidence, and metastasis This method had some issues, including learning process vibration, sluggish convergence, a network easily constrained to minima, and problem complexity.

#### 4.1.2. Naïve Bayes

The method proposed by [133] relies on Bayesian theory and is primarily appropriate when the input size is huge. Despite its simplicity, it can regularly outperform the categorization approach. This study utilized a 1018 CT scan of the OC from the UCI repository, which is the biggest publicly accessible library of information related to cancer screening. It achieved an accuracy rate of 85.71%; however, CNN performed better. Using contrast-enhanced CT imaging, the nodal state of oropharyngeal SCC and oral SCC was evaluated. With the receiver operating characteristic (ROC) set to 0.857, the NB’s bagging obtained a maximum accuracy of 92.9%. Romeo et al. [134] showed the conditional dependence of various variables, which was graphically represented by Bayesian networks. Probabilities are easier to observe, understand, and compare with other random variables when the information is presented graphically. This model’s drawback is that it struggles with highly dimensional data. For example, the Bayesian network in the study by [135] produced good validity measurements whenever data such as genetic traits and clinical imaging were inputted into the network.

#### 4.1.3. Decision Tree

To study prior OC instances, DTs and ANNs were used, and their effectiveness was contrasted with that of LR in the study by the authors in [136]. An association rule mining-based technique for cancer detection and prevention was used [9], which provided an overview of the many techniques employed by researchers to classify OC detection during the earlier stages. The DT classifier had the best performance (76% accuracy), followed by the LR model (60% accuracy), whereas the RF and KNN models performed poorly in comparison [137]. It is worth remembering that not all ML algorithms make sense. For instance, the outputs produced by SVM and an ANN are nonlinear as well as puzzling. In this regard, this model makes the collection of classification rules visible. Physicians frequently lack confidence in the outputs generated by the clinical decision support systems since the algorithm’s operation is uncertain about how it determines the classification result. Promoting the transparency of these methods in this regard is crucial since it can help make it easier for them to be applied in the real world [17]. Overall, the reliability of ML models for predicting the chances of an OC recurrence ranged from 64 to 100%. Two DT-based models demonstrated an exceptional predictive performance [138,139]. The top features (such as variables) that were indicative of oral cancer survival time were identified using DT regression to find the best predictors, and comparative variable relevance scores were generated. As DT regression does not demand a linear relationship between characteristics and the target value, it is the right method for this application [140].

The authors made use of the oral leukoplakia data set [141]. The correlation coefficient was used to identify the features after the data were modified to make it more uniform. The obtained attributes were categorized using random forests and DTs, and they were contrasted with other well-known popular classification methods such as MLP, KNN, SVM, and logistic regression. The experimental investigation found that RF and DT could successfully categorize oral cancer according to its different phases. As a result, distinct OC could be classified. To build the internal nodes and root nodes, the best class among all possible classifications in the descriptive data was chosen. The categories of the training part of the data could then be identified. The C4.5 algorithm was used most frequently among the existing DT algorithms [142].

#### 4.1.4. K-Nearest Neighbor

The KNN algorithm was used by the authors in [143] to offer important insights into how clinical factors can relate to one another including age, mutations, modified genes, and their effect on cancer patients’ length of survival. The objectives of this study were to: (i) Identify the contributing clinical factors to the survival time of the OC patients; (ii) Produce a clinical summary, and model an ML-based survival time prediction; (iii) Base the results on the model performance; (iv) Comprehend the connection between diagnostic factors for survival rates and phases of OC; and (v) Affirm the results using MAE and F-scores. As far as the author could tell, these elements had not been examined in previous research studies to determine how they might affect survival time. Additionally, one of the many stages of OC was identified in each patient’s medical file [143]. In [133], the outcome in the KNN rankings was considered as a group member. The object was allocated to the closest group of its neighbors if K = 1. When it focused on UCI, it had the lowest accuracy. Based on pathological and clinical statistics, Chui et al. [144] predicted the likelihood of developing cancer, evaluated KNN, SVM, BDT, and linear regression (LR) models, and found that BDT was the ideal model. The KNN model had an accuracy of 69.36% (AUC 0.71), a 35.11% sensitivity, and a 85.56% specificity when using bivariate analysis.

#### 4.1.5. K-means Clustering

The most fundamental, iterative, and unexpected learning algorithms are K-means. In this algorithm, every data point is compared to every center, and to group the results, k-means grouping is employed [145]. There are a variety of feature selection methods available, including Pearson correlation, correlation co-efficient, and principal component analysis. Finding the ideal number of characteristics from OC data is the goal of feature selection to develop a more practical and smaller model for OC forecasting. To find prospective OC patients, the research study carried out by the author in [146] used techniques that involved data mining techniques such as clustering and classification. Additionally, it utilized a genetic approach to choose features for OC [18]. The distinctions between the indications displayed in previous cases when patients died from or survived OC were determined using an integrated methodology [29] that integrated the clustering and classification approaches [132].

#### 4.1.6. Random Forest

Using a random forest classification method, the authors in [147] used Fourier transform infrared spectroscopy to identify normal stroma, malignant epithelium, normal epithelium, and cancer-associated stroma in stained histological samples acquired from the pathologist on conventional glass slides. The random forest tree classifier was used to implement the classification of pearl and keratin areas using Gabor texture information. Subepithelial layers, epithelial layers, and keratin pearl detection were segmented in the findings of the CAD method, which can be used for grading OSCC and screening for oral precancerous lesions. This would allow doctors to make quick, accurate diagnoses without bias. However, the algorithm created by the researchers is valuable in relation to the this research because it has been shown to reduce processing times [148].

#### 4.1.7. Support Vector Machine

Since an SVM is a statistical algorithm for reducing risk, it is commonly employed for OC risk prediction. James et al. [149] used an SVM model and ANN to elucidate picture attributes of OCT images taken from benign and healthy oral mucosa, and malignant tumors. In the modes of CNN, the SVM algorithm takes the place of the last layers. An SVM combines the retrieved deep features of ResNet-18 and AlexNet and uses them to detect problems with high precision and little training time. The identification of questionable areas in oral images has been made possible by a variety of image processing algorithms. Deepak Kumar et al. [150] referenced the utilization of hyperspectral imaging in order to detect cancer over other medical imaging techniques. They contrasted several imaging techniques for detecting cancer and discussed the advantages of hyperspectral images. HSI can therefore be employed by medical professionals for classification. It uses a self-organizing map structure that can be used for categorization. To determine the stages of OC patients, an SVM-based analytical model with the hybrid feature selection strategy, linear forward selection, and a hybrid correlation evaluator was created. The authors in [151,152] reviewed the prognostic and diagnostic applications of ML techniques in OSCC and brought attention to physicians’ restrictions and issues regarding the use of ML-based models in routine clinical practice. SVM was found to be the most extensively utilized ML algorithm for OC prognosis/diagnosis by far, despite the advantages of other techniques. This was also mentioned in a study that looked at AI techniques, alongside neck and head cancer genomic data [153]. A further study found that the most effective algorithm for forecasting OC survival rates was the support vector machine [154].

#### 4.1.8. Ensemble Models

Ensemble learning combines various distinct models to enhance generalization. DL models’ multilayer processing architectures currently surpasses shallow or conventional classification techniques with respect to precision and throughput. In a previous study combining both DL models and ensemble learning, the final model performed better in terms of generalization [155]. There was no pattern on the particular superior algorithm for predicting such an outcome, but ensemble learning-based classifiers outperformed other supervised methods. The mean specificity and sensitivity of the nodal metastasis classifiers were approximately 0.80 and 0.85, respectively, indicating good to outstanding accuracy [19]. Demographic factors, cytological traits, molecular profiles, and the utilization of supplementary testing were just a few of the features used to train models in the base research. The RF ensemble algorithm was the preferred task performance model in two of the three studies with external approval [156,157]. Nanditha et al. [158] created an automated system to evaluate oral lesions with the help of DL techniques. In this study, an ensemble DL approach integrated Resnet-50’s and the visual geometry group (VGG)-16. The expanded data set of oral lesion images was employed to train this strategy. In terms of implementing the classifier of oral pictures, this method was contrasted with other widely used DL techniques.

RF, SVMs, DTs, GBDT, and KNN were included as classification methods in the collection of several DL algorithms in [159]. Then, this ensemble classification strategy was applied to three sets of cancer datasets. According to the authors in [160], this strategy proved effective in making up for the drawbacks of each categorization method by using a combination. A classification accuracy of 95.60% was attained, surpassing the performance of each classifier utilized separately. Additionally, a regularized ensemble architecture was presented by the authors in [161] to address the issues in the earlier systems, with multi-class learning and unbalanced training data [162]. The ensemble algorithms outperformed an SVM, but only because of their ability to combine several algorithms with essentially the same bias and then combine the results generated to lower variance. Interestingly, there were no distinct difference between the ensemble methods with lowered parameters found in the PFI analysis and the algorithms without PFI in terms of the overall accuracies attained. As a result, it is possible to effectively control the costs and resources involved in obtaining multiple characteristics. Ensemble algorithms, as well as non-ensemble algorithms such as the NBs (a strategy based on the depth of invasion), and SVM outperformed other ensemble approaches in this study. The ensemble machine methods have to be taken into account in medical applications. Clinical early stage OC outcomes are currently difficult for clinicians to evaluate. Knowing how patients might be classified into high-risk or low-risk categories using ML technologies can assist physicians in making more informed decisions regarding their clinical practice [138].

#### 4.1.9. Summary of the ML Model

**Figure 9:**
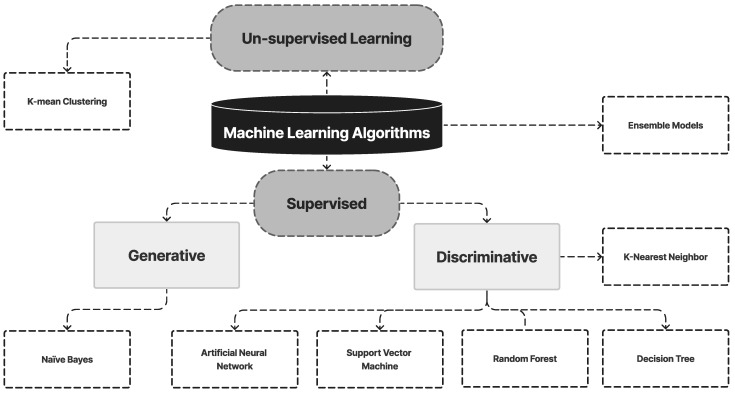
ML Models for Oral Cancer Diagnosis used in this review.

ML is a subset of AI that enables computers to learn from data and make predictions or decisions without being explicitly programmed. It has been applied in the field of oral cancer to improve the accuracy and efficiency of diagnostic tools, identify new biomarkers, and predict patient outcomes. One example of ML application in oral cancer is the analysis of digital images of the oral cavity to detect early signs of oral cancer. ML algorithms such as K-means clustering, random forest, an ANN, and SVMs have been used to analyze images of the oral cavity, such as those from X-rays, CT scans, and photographs. These algorithms have been trained on large datasets of oral cancer images and have been shown to achieve high accuracy in detecting oral cancer and distinguishing between benign and malignant lesions. Figure 9 illustrates the ML models for OC diagnosis used in this review. Table 3 presents a summary of studies on ML models for Oral Cancer diagnosis.

#### 4.1.10. Limitations of the ML Model

It is worth mentioning that while ML has great potential to improve the diagnosis, treatment, and prognosis prediction of oral cancer, it is still in the early stages of development and requires further research and validation before it can be widely used.

### 4.2. Deep Learning Techniques

#### 4.2.1. Recurrent Neural Networks

Oral malignant development is a challenging issue for people, due to its aggressive nature and high visibility. Clinical evaluation by qualified healthcare experts is time-consuming process. Differentiating the evidence at the outset usually facilitates more effective corrective actions. A very precise configuration of any recognition system depends on the proper operation of a few selected components, such as improved derivation, order, and quick and high-resolution cameras. RNNs are a subclass of neural networks that are useful for displaying progression information. RNNs, which were derived from feedforward networks, exhibit behavior that is quite like that of human personalities. Redundant brain networks are able to create visionary conclusions in progressive data which other algorithms cannot. The authors in [169] concluded that this technology allows for the early diagnosis of oral malignant illness.

#### 4.2.2. Deep Autoencoder

An autoencoder can combine the features and data from several omics sources thanks to its architecture [170,171]. Zhang et al. [172] combined multiomic cancer data using a variational autoencoder. The resulting model was then utilized to create a pan-cancer classification analysis, which achieved an average precision of 97.49%. Variational autoencoders were used by Simidjievski et al. [173] to investigate the various construction techniques, designs, and architecture construction techniques for multiomic data integration approaches. They showed that autoencoders can act as an effective method for the representation of the data, along with the creation of reliable diagnostics. Compared with other techniques, this enabled the discovery of more favorable genes linked to such forms of cancer [174]. The algorithm needs to be trained using a less sophisticated neural network utilizing the right collection of input datasets to effectively categorize photos of OC. The overfitting issue, which prevents the creation of the precise feature map, has been observed in research on this network. In an attempt to overcome this, Antonio et al. [175] built an autoencoder architecture for extracting distinguishing information from the input data; although, it still has reconstruction issues. As a result, there is still room for improvement in the existing literature.

To assess local phenotypic features and to know where they are located in the tissue, the authors in [175] experimented with utilizing a distinct network design, and the network was widened to handle more problematic input photos. The state-of-the-art solution was enhanced by the authors in [176]. They suggested using deep convolutional autoencoders to gather the distinctive features from the supplied inputs as a solution. To analyze larger pathological pictures and identify the transcriptome subtypes, they also constructed a sample based on one classification reducer and three autoencoders. For larger tissue input, it can be challenging to distinguish between the statistical distribution of cellular characteristics [177]. The convolution filter scans the input data and samples using a max pooling layer. Afterwards, it passes to the layer of encoding, which performs deconvolution as well as de-pooling to obtain the optimized picture input. This facilitates the extraction of higher-order, more complicated picture information. A high SP value of 81.35% was attained using the auto-encoder. For prognosis, treatment planning, and the provision of detailed feedback from specialists concerning the nature of oral lesions, precise screening of OC biopsy pictures is crucial. In this article, the authors proposed an automated and efficient computer method for identifying both normal cells and the early stages of OC using histopathology pictures. Compared with other approaches now in use, the proposed system yielded the best outcomes, according to a qualitative and comparative analysis. We think that this plan is quick, affordable, and precise. As a result, it can be used by clinicians as an extra diagnostic tool during routine clinical examinations [178].

#### 4.2.3. Deep Neural Network

A novel strategy for combining the bounding box annotation from various doctors has been highlighted in the literature [179]. DNN was also used to build automated systems that can tackle this challenge by producing patterns. Two DL-based CV techniques were taken into consideration for the automatic recognition and classification of oral lesions because the primary data gathered in the case can be used. A DL method for computer-aided and automated OC diagnosis was developed which took the patient’s hyperspectral images into account [180]. To forecast the patients’ overall and progression-free survival, Scientists created a DNN [181]. This network used the clinical information, tumor features, and therapy information of the patients as the inputs. The DNN model performed better than the COX model when the proposed model was compared to the latter. A DNN model called DeepSurv wascreated that used baseline patient data to forecast the impact of covariates on the hazard rate [182]. ML techniques were examined to predict the incidence of OC in OPL patients [183]. The results demonstrated that the FD with DNN had a high accuracy rate when compared with other classification techniques. Fisher’s discriminate analysis uses the statistical properties of the data to distinguish between groups of data. The simplicity of implementation makes it suitable for medical applications.

When OCSCC, non-OCSCC oral illnesses, and normal oral mucosal tissue are segregated into three clusters, we can see that the points of the same lesion class were collected into one cluster with the same hue. However, the proposed algorithm was still unable to differentiate some aesthetically perplexing situations, such as epulis. The effectiveness of a much larger, more diversified training dataset will be examined in upcoming clinical trials as a potential remedy to this problem [184]. Researchers assessed the Bayesian DNN’s intraoral cancer image classification accuracy. The outcomes were contrasted with those of a traditional network with typical loss. The efficiency of the traditional network with standard loss was 85.1%, compared with the BDNN’s accuracy of 85.6%. The outcome demonstrates a good classification performance for the Bayesian network and implies that ensemble learning slightly enhanced performance without sacrificing accuracy for the DNN [185].

#### 4.2.4. Deep Belief Network

For a large image data set, DL enables accurate categorization and outperforms the human level of classification [186]. To equal the performance of the experts in this research on the classification of benign and malignant images, the authors in [150] designed and created a partitioned CNN. Since the DL algorithm requires fewer features to be trained and offers more effective learning tools, the authors in [187] contrasted the classification performance with more established methods such as SVM and DBN.

Prior to surgical intervention, radiological imaging techniques including computed tomography (CT) and magnetic MRI can calculate the size and extent of an OSCC. However, these methods are not sensitive enough to find precancerous lesions. Hyperspectral imaging (HSI) and optical fluorescence imaging (OFI), two adjuvant clinical imaging techniques that have been used to help diagnose OC, have been used to overcome this obstacle. These images have the potential to be examined using complex algorithms [188].

#### 4.2.5. Deep Convolutional Neural Network

In [6], a huge number of OSCC and non-OSCC images were utilized to train the model. Other CNN models have been combined with the VGG16 and ResNet50 CNN models. CNN and random forest models were used by Das et al. [164]. The random forest model correctly identified keratin pearls with 96.88 percent accuracy, while the CNN model correctly segmented keratin areas with 98.05 percent accuracy. Another option was CNN, which had an accuracy rate of 97.5 percent [11]. Following surgical excision, deep CNNs were shown to have high sensitivity and specificity in differentiating between tongue SCC and normal tissues assessed by fiber-optic Raman spectroscopy [189]. Using Inception v3 CNN, the authors in [15] achieved a classification accuracy of 87.02%. The researcher’s goal was to evaluate cutting-edge automated approaches for diagnosing OSCC using crisp images and CNN techniques. Its main areas of interest included classification, learning, data, and picture searching. The development of OC in OPL patients was predicted by the researchers using genetic data and ML techniques. The researchers used an SVM, multi-layer perceptron, a minimally invasive technique, and a DNN to assess the progression of OC in people with a history of OPL [183]. Various algorithms and strategies were used in a current DL study to improve feature extraction and image analysis. It is important to train the algorithm using a less sophisticated neural network utilizing the right collection of input datasets to effectively categorize photos of OC.

#### 4.2.6. Deep Generative Models

A computer-assisted method to diagnose oral pre-cancer/cancer utilizing oral exfoliative cytology was suggested by Chatterjee et al. [190]. For the diagnosis of OC, they used statistical variables such as morphology, strength, color, texture, and distribution. Using a random forest classifier, they achieved a maximum recall accuracy of 94.58%. AI demonstrated a strong performance in predicting the debonding probability of CAD-CAM CR crowns using 3D STL models scanned from patients. Dental professionals could benefit from this technology during or following restorative operations as well as in other challenging situations, such as root or die fractures [191]. The effectiveness of image-processing methods in the detection of cancer was assessed by Tanriver et al. [192]. A two-stage DL model was proposed using a second-stage classifier network to detect OC and classify the detected area into three categories of benign lesion, mouth cancer, and possible malignant carcinoma.

#### 4.2.7. Deep Boltzmann Machine

Pre- and post-cancerous lesions were detected using this method with normal jobs with accuracy rates of 81.92% and 86.52%. However, SVM required additional processing resources. The authors also classified the same hyperspectral database using DBM. The higher-level feature extraction investigated utilizing the minimal spanning tree method organized the feature spatial ordering. In this instance, the authors in [193] achieved an accuracy of 84.50% and 89.52% for two different tasks. This demonstrates that we can improve diagnostic performance by combining base classifier feature extraction. The identical two hypercube data sets yielded accuracy results of 91.55% and 94.75%.

A classification of the microscopic pictures of SCC from histopathology slides into different categories of OSCC was carried out. When carrying out the categorization, the texture characteristics of the photos were taken into account. The methods utilized for DWT are effective tools for image and signal processing. DWT is better suited for the extraction of features from images because of its simplicity and low computational complexity. One of the popular feature extractors is GLCM. The DBM classifier was used to categorize the model. For an effective comparison of this method, computational time was also taken into consideration. Accuracy and dependability were always given great priority because diagnosing cancer is a difficult and delicate task. The suggested system’s output was contrasted with a previous ANN classification study. This study had 93.37% accuracy compared to the 87.92% accuracy achieved in a previous study, demonstrating the superiority of the proposed model [194]. To categorize oral and neck cells using hyperspectral images, a mixed architecture made up of DBM and SVM was developed. High-level characteristics were extracted using the minimum spanning tree method. The model’s accuracy ranged from 91.55% to 94.75% [193].

#### 4.2.8. Deep Reinforcement Learning

Martino et al. [195] divided oral lesion whole slide images (WSI) into three groups using a variety of DL architectures, including U-Net, U-Net with VGG16 encoder, SegNet, and U-Net with ResNet50 encoder (carcinoma, non-carcinoma, and non-tissue). It has been demonstrated that a deeper network, such U-Net upgraded with ResNet50 acts as an encoder and is more reliable than the initial U-Net. Authors recently used extracted features to carry out binary classification on images of oral disease using VGG16, Inception V3, and Resnet50, which were each separately tweaked [196].

#### 4.2.9. Extreme Learning Machine

Extreme Learning Machines (ELM), a feedforward ANN, and a probabilistic model (Gaussian) have all been used by Rajaguru et al. [4] to categorize tumor cells. In this case, 75 photos were examined. GMM offered a better result than MLP for every stage, with an average accuracy of 94.18%. The performance of each model was evaluated, and ELM produced superior outcomes [197,198,199,200]. For the examination of OC, ELM was utilized as a post-classifier, and the performance was compared to that of GMM and MLP. This research created a novel model employing DNN technology to identify dental abnormalities in X-ray images and categorize dental X-ray images as normal or deteriorated [201]. The ELM is a forward neural network for single-layer or multilayer hidden terminal classification and functional training when the hidden node restrictions do not need to be established. As it includes a predetermined network design, ELM is simple to use and has a quick learning curve [133].

#### 4.2.10. Summary of DL Models

**Figure 10:**
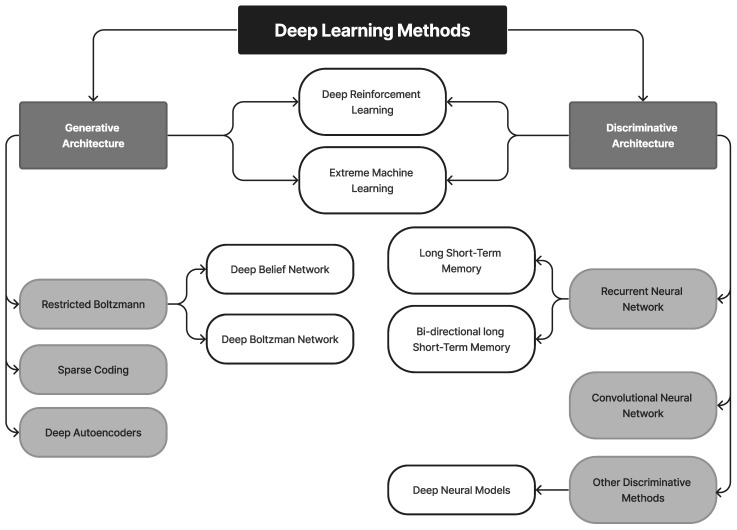
DL Models for Oral Cancer Diagnosis used in this review.

DL algorithms have been used to develop models for the diagnosis of oral cancer. These algorithms, based on deep CNNs, are effective in analyzing medical images to detect signs of oral cancer. One approach is to use deep CNNs to analyze oral cancer images and train the model to recognize the characteristic features of oral cancer, such as lesions or tumors. Another approach is to use DL algorithms to analyze oral cancer images in conjunction with other types of data, such as patient demographics and medical history, to improve the diagnosis by taking into account additional information about the patient. Other types of DL algorithms, such as recurrent neural networks (RNNs), autoencoders, and generative adversarial networks (GANs), have also been used for oral cancer diagnosis. RNNs are useful for analyzing sequential data, autoencoders are used for unsupervised feature learning, and GANs are used for generating synthetic images that can be used to augment the training dataset. Overall, deep learning algorithms are effective in the diagnosis of oral cancer and have the potential to improve the accuracy and efficiency of the diagnostic process; however, further research is needed to fully understand their potential and overcome the challenges associated with their development and use. Figure 10 shows the DL models for OC diagnosis used in this review. Table 4 shows a summary of studies on DL techniques for OC diagnosis.

#### 4.2.11. Limitation of DL Models

DL is a powerful technique for ML, but it also has several limitations that need to be addressed: The DL models can be limited by the current understanding of the disease; if the underlying biology of the disease is not well understood, the model may not be able to accurately diagnose the disease. Furthermore, the data used to train the model may not be diverse enough to be generalizable to different population groups, which can lead to poor performance in certain population groups. A lack of generalization, bias, overfitting and computational requirements are other common challenges that need to be addressed for image-based OC diagnosis. In image-based disease diagnosis, the explainability of the model is very important, as it may be complex and difficult to interpret sometimes. Overall, while DL has shown great promise in various applications, it is important to consider these limitations and address them in the design, development, and deployment of DL models.

## 5. Open Challenges

There have been many difficulties in the creation of the ML model. However, there has been a substantial advancement in ML applications in recent studies, such as the annotation of a two-dimensional landmarks and the diagnosis of oral illnesses. Some of these technologies have attained accuracy levels that are as high as the current gold standard or even higher. In oral medicine, the ability of ML to analyze images is particularly important. Nevertheless, numerous applications are still in their infancy and are not yet ready for clinical use. Considering data shortages, attempts can be made to transform learning models. For instance, a complicated, essential model could be divided into several modularized structures, older ML models could be modified to handle a variety of accessible data, or a method that amalgamates manual work with computer software could be adopted. This line of inquiry will also be aided by the future generation of superior medical imaging data sets. This issue will also be solved by brand-new weak and semi-supervised techniques. The operational cost must be considered in addition to the prototype model. In general, ML has a promising future as a means of enhancing clinical effectiveness and diagnostic precision [138]. The difficulties surrounding the explicability of ML models is one of the clinical concerns. For doctors to explain the performance measures and how the system arrived at the prognostication, the models must be practical and simple to use [210,211,212]. Other concerns of professionals include how these potentially revolutionary tools would alter the interactions between patients and clinicians [210].

Some of the challenges include job competition [212]. Misunderstandings concerning the scope of AI in medical diagnostics represent a large proportion of the existing difficulties in translating ML models for use in routine clinical practice. However, creating a solid model relies heavily on the expertise of the ML experts and the caliber of the data utilized in the analysis. As a result, to create a high-quality model, the data quality used for model training must be the finest available and well-structured [210,213]. Most of the included papers did not assess the difficulties associated with integrating ML models into routine clinical procedures, which is the primary drawback of this systematic review. As a result, from the research that was included, no potential remedies could be deduced. The accuracy and quality of the data that DL algorithms use as input are crucial. It is possible that standard medical record data, which now lacks improved histopathological imaging, biomarker studies, and genetic profiling, will eventually be insufficient for predictive analyses. DNNs may one day be more effectively used to examine the intrinsically complicated biology of tumors since they enable multiple-layer extraction of progressively complex data and resemble human decisions.

### 5.1. Precision Medicine

Precision medicine is an approach to healthcare that uses individualized treatment plans based on a patient’s unique characteristics and medical history. AI-based disease diagnosis can play a key role in precision medicine by providing individualized and accurate diagnoses of oral cancer. In the near future, we are expecting the implementation of a personalized medical database for everyone. AI-based disease diagnosis models can be trained using a patient’s medical data, such as imaging and genetic information, to create personalized diagnostic models. These models can improve the accuracy of oral cancer diagnosis by considering a patient’s unique characteristics. It can also be used to monitor patients with oral cancer to track the progression of the disease and adjust treatment plans accordingly or assess a patient’s risk of oral cancer based on their medical history, lifestyle factors, and genetic information. Despite the claimed potential advantages of DL techniques for precision medicine, several significant obstacles still need to be overcome. First and foremost, the quality of the data (such as imaging or pathology data) to be used in the research must be extremely good. To achieve this, specialists in ML and pathologists, for example, should agree on a standard resolution for the images. This will guarantee that the clarity is high enough to capture the tumor’s microenvironment and histological characteristics. AI-based disease diagnosis can play a key role in precision medicine for oral cancer by providing an individualized and accurate diagnosis, identifying patients at high risk of the disease, and tracking the progression of the disease. This can help to improve patient outcomes and tailor treatment plans to the individual patient. To obtain images that are useful in the DL analysis and eventually for precision medicine objectives, there should be a uniform procedure for both the radiological and pathological images acquired.

#### 5.1.1. Using Appropriate Datasets

The generalizability of the created models is crucial. Therefore, a sizable dataset of excellent image quality should be used to train the model. The manipulation of real data to produce appropriately diverse data for DL training is another method for producing a generalizable model. The model’s generalizability guarantees that it will produce a reliable performance indicator over the whole proposed target population. External validation may guarantee that the model will function as intended and without bias; however, the model must perform well for a variety of populations. It is interesting to note that research is currently being carried out to create a generalizable model. The very tiny patient and data pool, the practically universal retrospective character of the investigations, and the potential for overfitting issues all contribute to these difficulties. Since most conventional statistical models presume the input data have a comparable structure and distributions, it is well recognized that irregular or missing sampling data in the healthcare sector presents a unique problem. Failure to adhere to these presumptions may result in learning issues and subpar model performance [214]. Less training data or class inequality in the training set are significant problems in medical image analysis [215]. Imputation or more sophisticated DL methods such as LSTM and RNN models are the usual solutions for missing feature issues [216,217]. Medical image analysis lacks rare, high-quality information that is identified as being publicly available. Most of the data sets discussed in this OC evaluation involved fewer than 100 patients. Despite the short training data sets, the articles in this [218] report demonstrate good performance in the various assignments. Cho et al. [219] highlighted that the more data we need, the more powerful the abstraction we want. Less training data or class inequality in the training set are significant problems in medical image analysis. Overfitting could occur if the right number of training samples are not employed. Although there is a chance of overfitting, data inequality can be improved by utilizing data augmentation to create additional atypical picture training samples [199,209]. A well-known difficulty with DL is the volume of data needed.

The way that this criterion skews the types of data resources used in AI research and the relative scarcity of some of the related data may be less well known. For instance, historical registry data gathered from routine clinical care or opensource datasets are used to provide enough input data in numerous investigations. The labels associated with the photos in these image repositories are hardly subject to quality control, leaving the DL model susceptible to errors and undetected biases. Inferences on generalizability to other groups are frequently not possible for these huge datasets (either because they are not collected or because of accessibility issues), which raises the likelihood of bias toward specific demographics. Since DL relies on large datasets, adding more data will dramatically enhance outcomes and enable the discovery of complex, generalizable patterns [220].

#### 5.1.2. Use of Bio-Inspired Computing Approaches

Bio-inspired computing approaches, such as evolutionary algorithms and neural networks, have been used in AI-based disease diagnosis to improve the performance of the models. Several bioinspired algorithms can be explored to improve AI-based disease diagnosis: evolutionary algorithms, artificial immune systems (AISs), artificial bee colony (ABC) algorithms, artificial fish swarm algorithm (AFSA), bio-inspired DL models, and ANNs. In contrast to the optimization algorithm, meta-heuristic algorithms aim to offer the best possible solution to the issue with the least amount of time and effort. These algorithms have demonstrated their effectiveness in numerous other disciplines. However, these techniques are not often utilized in survival analysis, and their accuracy can be increased by combining them with ML algorithms. Wang et al. [221] coupled particle swarm optimization and the synthetic minority over-sampling technique (SMOTE) to predict the 5-year cancer survival among breast cancer patients. Overall, bio-inspired computing approaches can improve the performance of AI-based disease diagnosis by optimizing the parameters of the models and incorporating the structure and function of biological systems into the design of the models.

#### 5.1.3. Difficulty in Achieving Accuracy

Despite the systematic and well-established methods for histopathologic evaluation, some medical diseases may present the physician with significant difficulties in reaching a reliable diagnosis. Firstly, suspicious lesions in an oral cavity exposed to carcinogens frequently cover a wide region or are dispersed in several locations. Multiple biopsies may be necessary if the mucosa has been impacted by field cancerization or if there are many lesions with different cancer-developing statuses. Nevertheless, despite the existence of symptomatic or asymptomatic lesions, many patients fear repeated biopsies because of their invasiveness. Several parameters need to be optimized to obtain maximum accuracy in real-life samples such as regularization, data balancing, augmentation, cross-validation, etc. Further transfer learning is also a powerful approach to achieve better detection accuracy.

#### 5.1.4. Choosing the Correct Features

Choosing the correct features is crucial for the model’s performance and accuracy as the features used in the training dataset are the inputs that the model will use to make predictions or classifications. Relevant and informative features should be included while avoiding irrelevant or non-specific ones. Additionally, the dimensionality of the feature set should be considered to prevent overfitting or underfitting. In survival analysis, choosing the appropriate feature set for prediction is a significant difficulty. Exploring the common qualities and hidden aspects should be prioritized because most studies employ multimodal data.

#### 5.1.5. Trustworthy AI

Transparency is important because it allows people to understand how the AI system works and how it makes decisions. Interpretability is important because it allows people to understand why the AI system made a particular decision. The model must perform as predicted. As a result, the model’s training phase mistakes should be as low as possible. To ensure that the model and, subsequently, the outcomes from these models are transparent, any type of error or malfunctioning, or breakdown of the model should be identified and be conspicuous. To ensure the model’s credibility, a potential data imbalance should be considered when creating the model. Similar rules for transparent reporting can be used to solve this problem [109,222]. The ML model used with these recommendations will be reliable and uphold the ethical criteria of clarity, authenticity, audibility, reliability, and recoverability, as well as the four cornerstones of medical ethics: autonomy, beneficence, and nonmaleficence. It is also important to involve a diverse group of stakeholders in the development and deployment of the model, including experts in AI, ethics, and the relevant domain, as well as representatives from affected communities to get unbiased and expected results.

#### 5.1.6. Data Privacy and Confidentiality

Data privacy and confidentiality are also major concerns in AI-based disease diagnosis systems. Some of the issues include:

- Data breaches: AI-based disease diagnosis systems store large amounts of sensitive patient data, making them a target for cyberattacks. A data breach could result in the unauthorized access or disclosure of patient information, which could lead to serious privacy violations.
- Data sharing: AI-based disease diagnosis systems often share patient data with other organizations, such as research institutions and other healthcare providers. This can raise concerns regarding the security and privacy of data, as well as the potential misuse of data.
- Data anonymization: AI-based disease diagnosis systems may use anonymized data to protect patient privacy. However, it is possible to re-identify patients from anonymized data, and there is a risk that the data could be used for unintended purposes.
- Data storage: AI-based disease diagnosis systems store large amounts of patient data. This data can be stored in multiple locations and can be vulnerable to hacking, data breaches, and data loss.
- Lack of transparency: AI-based disease diagnosis systems may lack transparency in the way they collect, store, and use patient data, which can make it difficult for patients to understand how their data are being used and control access to their data.
- Bias and discrimination: AI models can be affected by bias and discrimination, which can lead to inaccurate or unreliable results, especially for a certain population group.

**Figure 11:**
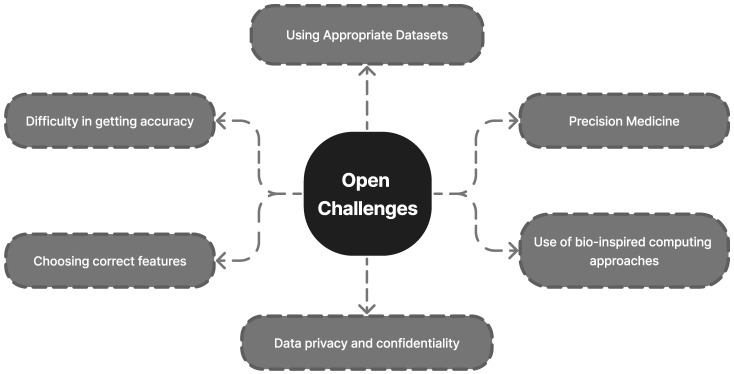
Open Challenges for Oral Cancer Diagnosis.

The privacy of healthcare data is the first of these ethical issues. Patient healthcare data are heavily utilized for creating ML models. Consequently, this poses issues with patient confidentiality and privacy. To address this issue, patients or other subjects must be informed about how their data are collected and used, to ensure informed consent, prevent unauthorized proprietary use of the data, and protect their privacy. The group of imaging researchers, experts, radiology executives, and informaticists must discuss how to handle these confidentiality problems and help pave the path for the development of solutions, as equitably and ethically as possible. However, data use agreements must be examined and agreed upon by the relevant parties. Additionally, a method (i.e., a clinical decision with cloud support that protects patient privacy concerning their data) can be used to keep patients’ data private. The possession of data debate, however, is outside of the remit of this study. Overall, data privacy and confidentiality are major concerns in AI-based disease diagnosis systems, and it is important to ensure that patient data are protected and used responsibly. This can be achieved by implementing strong security measures, adhering to data privacy and security standards, and providing transparency and control for patients over their data. Figure 11 illustrates the open challenges or unresolved obstacles in utilizing AI-based methods for diagnosing oral cancer. Table 5 presents the open challenges for Oral Cancer diagnosis.

## 6. Limitations of This Review

Only data from publications in the Scopus and PubMed databases were used in this review. As a result, the constraints of those articles had an impact on the review’s level of information. These limitations included a lack of research concerning coordinating diagnostic methods and biopsy research findings, the difficulty in achieving an automated processes, the need for storage infrastructure, building and training AI models, the limited amount of available data, image quality, the retrospective collection of data, the comparison of images taking into account that healthy tissue could be present with mucosal alterations, imbalances in the data used to train the model, the possibility of missing data, and more. When the collection of written text is small, the model is overfitted, which causes erroneous cancer stage categorization and survival time prediction. Additionally, feature choice is a critical component of integrity. An operator’s basic inaccuracy in entering data one row down can result in inconsistent data, which can eventually accumulate to many errors in the later assessment step. Factors such as measurability, pathological evaluations, and relevance must be considered when choosing crucial elements from the dataset; the present study fell short in this regard.

This study has several drawbacks. There are several other general limitations of the meta-analysis that cannot be avoided such as heterogeneity, publication bias, quality of the studies, data complexity, etc. In addition, there were also certain drawbacks specific to this paper. First, our findings were based on data that was collated from studies using various imaging methods. The outcomes were heterogeneous as a result and each imaging tool’s sensitivity was examined individually. However, the work is significant as the first meta-analysis for evaluating the precision of AI-based image analysis. Second, even when using the same imaging instrument, the accuracy of the diagnosis may be impacted by the quality of the devices used in each study and variations in the procedures. The variety of oral lesions may not be accurately represented by the photos used to train the AI algorithm. Third, the interpretation of the findings was constrained by the complete lack of prospective trials comparing conventional examination to AI imaging diagnosis. It is our responsibility to research this across a range of clinical disciplines to prepare for a day when AI-assisted healthcare will be effective. Another drawback was that the dataset included some low-resolution photos. As the dataset size increases, we will impose limitations on the allowable image resolution, encouraging the production of high-quality data.

## 7. Future Research Directions

The existing restriction of prognostic variables in terms of sociodemographic and clinicopathological factors is a barrier to AI-based outcome prediction in OC healthcare. Hence, there are several areas of research that can be pursued to further develop AI-based cancer diagnosis.

### 7.1. Integration with Other Diagnostic Tools

An AI-based cancer diagnosis should be integrated with other diagnostic tools such as biopsy and laboratory tests to improve the accuracy of diagnosis. Research can be focused on developing methods to combine the results of AI-based diagnosis with those of other diagnostic tools to improve diagnostic accuracy. The application of AI to the prognosis and diagnosis of diseases has advanced in recent years. Previous research has demonstrated that ML and DL yield reliable results for OC detection. It avoids unintentional errors and helps professionals with diagnostic procedures. However, compared to ML, earlier research using DL (neural networks) was more accurate in the early detection of OC. AI offers the chance to create novel approaches that work in conjunction with established methods to increase the accuracy of OC and OPMD identification, as well as the ability to forecast the progression of pre-cancerous and cancerous lesions using the data collected. Future studies might look towards developing data fusion algorithms that combine different modalities, such as histological, clinical, radiological, and molecular evaluations, to support early illness diagnosis and result prediction.

### 7.2. Handling Missing Data and Uncertainty

Research can be focused on developing AI-based methods that can handle missing data and uncertainty that are common in medical images and data. While these are undoubtedly the most accessible data types, they may nevertheless be prone to inaccurate data input and aggregation during data collection, particularly if data are not recorded prospectively. Essential but underutilized classes of predictive variables for intelligent learning are molecular prognostic markers.

### 7.3. Personalized Medicine

AI can be used to analyze large amounts of patient data, including genetic and molecular data, to identify personalized treatment plans for cancer patients. Research can be focused on developing AI-based methods to identify the most effective treatment options for individual patients based on their specific characteristics.

### 7.4. Deep Learning Algorithm

Research can be focused on developing more advanced DL algorithms that can improve the accuracy and efficiency of AI-based cancer diagnosis. This can include developing algorithms that can analyze multiple types of medical images and data, such as X-rays, CT scans, and MRI scans, to improve the detection of cancer. There is a need to develop hybrid neural network models to improve effectiveness and efficiency. Using various strategies for augmentation and fine-tuning, it will be feasible to collect additional photographs to expand the dataset and increase reliability. To enhance the accuracy of the findings from the models, the key objective should be to build a semantic segmentation for choosing the lesion zone from an input image.

### 7.5. Real-Time Analysis

Research can be focused on developing AI-based methods that can analyze medical images and data in real time, which can improve the efficiency and accuracy of diagnosis. We should work to make more multidimensional clinical and biological data available for DL techniques. This is crucial to further boost the number of databases and, consequently, the advantages of applying AI methods, which appears to be an exciting new tool for the precise treatment of OC.

### 7.6. Explainable AI

As AI models become more complex, it becomes increasingly difficult to understand how they reach their conclusions. This can be problematic when it comes to medical diagnoses. Therefore, research can be focused on developing explainable AI models for cancer diagnosis, so that the reasoning behind the diagnosis can be understood by both the AI and the clinician.

### 7.7. Automated Diagnosis

**Figure 12:**
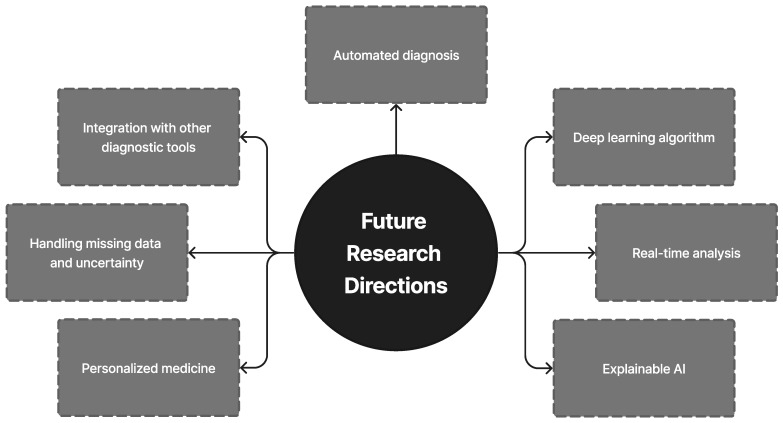
Future Research Directions for Oral Cancer Diagnosis.

Research can be focused on developing AI-based systems that can automatically diagnose oral cancer, which can help to improve the accuracy and efficiency of diagnosis. Powerful AI methods that can manage the available clinical dataset and deliver respectable prediction performance should be developed with focus. Additionally, the essential frameworks for implementing these models into regular clinical practices should be created. Failure to take into consideration all of these factors could result in an accumulation of studies on the potential of DL methodology in accurate patient outcome prediction but without any real benefit to a specific OC patient. This is required prior to their implementation as a clinical support system to offer the practitioner a second view in everyday practice. Overall, AI has the potential to revolutionize the field of OC diagnosis and improve patient outcomes, but it is important to continue to research and develop AI-based methods to improve their accuracy, efficiency, and applicability. Figure 12 illustrates the future research directions of oral cancer diagnosis, utilizing AI-based methods.

## 8. Conclusions

AI has been applied in the field of oral cancer diagnosis to improve the accuracy and efficiency of diagnostic tools. AI-based methods can be used to analyze images of the oral cavity and to detect early signs of oral cancer. Studies have shown that AI-based methods can achieve high accuracy in detecting oral cancer and that they can also be used to distinguish between benign and malignant lesions. Risk assessments can be produced by combining micromorphological features with geographic information, risk variables, different signal intensities, and patterns. It has already been proven that AI-based OC diagnosis is possible and feasible. Overall, early diagnosis of oral cancer is important for better patient outcomes; however, this still presents a challenge for healthcare professionals due to the lack of symptoms in the early stages of the disease, difficulty in visualizing the oral cavity, limited resources, lack of awareness, limited diagnostic tools, and difficulty in distinguishing benign from malignant lesions. According to recent systematic reviews, Asia had the greatest global prevalence of lip and oral cancer. Consequently, most of the investigations have concentrated on this area. For OC screening and detection, a variety of AI-powered imaging techniques have been explored. For instance, clinical images have been used in several studies to show how suspected OSCC lesions may be simply and automatically distinguished using algorithms [223,224]. It is established that for early OC diagnosis, DL is more accurate than supervised ML. AI has been applied to cancer risk assessment, lymph node metastasis prediction, and cancer prognosis. Early detection and prevention of OSCC depend greatly on the ability to forecast the malignant transition of OPMDs. A small number of studies have employed optical coherence tomography (OCT) to provide diagnoses using AI. The need for users to receive training to read OCT pictures would be eliminated by adding a diagnostic algorithm to the existing OCT system. AI algorithms combined with the knowledge of skilled pathologists could produce better results with fewer diagnostic blunders. One example of an AI-based method is deep learning, which is a type of machine learning that uses neural networks to analyze images.

DL algorithms have been trained on large datasets of oral cancer images to detect abnormalities such as tumors and precancerous lesions. These algorithms can be used to analyze images of the oral cavity in real time and to provide a diagnosis within seconds. Another example of an AI-based method is a computer-aided diagnosis (CAD) system using software that integrates AI algorithms and image analysis techniques to assist radiologists and clinicians in diagnosing oral cancer. This software can help radiologists and clinicians to identify malignant tumors and precancerous lesions in the oral cavity, helping to improve the accuracy of diagnosis and the detection of early-stage cancer. In addition, AI-based methods can be used to analyze large datasets of genetic and molecular data to identify new biomarkers for oral cancer. Researchers have used AI algorithms to identify gene signatures that are associated with oral cancer, which could be used to improve diagnosis and treatment planning. In conclusion, cancer diagnosis and AI have a strong link as technology has played an important role in improving the accuracy and efficiency of cancer diagnosis. AI-based methods, such as DL and CAD systems, have been developed to analyze medical images and large datasets of genetic and molecular data to identify new biomarkers for cancer. These technologies can aid in early detection and diagnosis and help in treatment planning. However, it is important to note that while AI has the potential to improve cancer diagnosis, it is still in the early stages of development and requires further research and validation before it can be widely adopted in clinical practice. In addition, there are several challenges with using ML for the diagnosis of cancer, including the availability of high-quality and diverse training data sets, the quality of the data used to train such as missing, inconsistent, or biased information, and overfitting and correct interpretability. However, with continuing efforts to improve the data quantity and quality, and improve the technology used, these problems can be solved. Additionally, it is important to remember that AI should be used in conjunction with other diagnostic tools, and not as a replacement for the expertise of healthcare professionals. With continued research and development, AI has the potential to revolutionize the field of cancer diagnosis and improve patient outcomes.

## Figures and Tables

**Table 1:** A list of the acronyms used in this article along with their full names.

Acronym	Definition
AC	Ameloblastic Carcinoma
AF	Ameloblastic Fibroma
AI	Artificial Intelligence
ANN	Artificial Neural Network
BCC	Basal-Cell-Based Lip Cancer
BDT	Boosted Decision Tree
BP	Back-Propagation
BSC	Basaloid Squamous Carcinoma
CAD	Computer-Aided Detection
CCOC	Clear Cell Odontogenic Carcinoma
CNN	Convolutional Neural Network
CT	Computed Tomography
DBM	Deep Boltzmann Machine
DT	Decision Tree
DL	Deep Learning
DNN	Deep Neural Network
FOM	Floor of Mouth
GCOC	Ghost cell odontogenic carcinoma
KNN	K-Nearest Neighbor
LOC	Lab-On-a-Chip
LSTM	Long Short-Term Memory
ML	Machine Learning
MRI	Magnetic Resonance Imaging
OC	Oral Cancer
OCS	Odontogenic Carcinosarcoma
OCT	Optical Coherence Tomography
OPMD	Oral Potentially Malignant Disorders
OSCC	Oral Squamous Cell Carcinoma
PIOC	Primary Intraosseous Carcinoma
PIOSCC	Primary intraosseous Squamous Cell Carcinoma
SCC	Squamous Cell Carcinoma
SCCOC	Squamous Cell Carcinoma of the Oral Cavity
SOC	Sclerosing Odontogenic Carcinoma
SVM	Super Vector Machine
VGG	Visual Geometry Group
WHO	World Health Organization

**Table 2:** Comparison with Prior Surveys/Comparison with Other Review Articles of a Similar Nature.

Reference	Year	One-Phrase Summary	ML	DL	OC	FD
Our paper	-	This review offers a thorough assessment of DL and ML models for diagnosing OC	H	H	H	H
[3]	2020	In order to lower the frequency of OC in India, this study emphasized the significance of early identification, adequate treatment, and prevention.	N	N	H	H
[26]	2021	This review concluded that it is essential to differentiate between malignant and benign cells while diagnosing OSCC. Additionally, it included a general summary of the elements of a delayed OC assessment.	L	M	L	N
[27]	2020	The results of SCCOC therapy in a newly published major series were presented in this study. It has been established that improved early detection techniques and understanding, as well as increased education concerning the risk factors connected to lifestyle choices, are essential for both the primary and secondary prevention of OC.	N	N	L	L
[28]	2012	The authors noted that men and people aged over 65 years had greater rates of lip cancer. The results demonstrated that SCC patients displayed typical clinical and epidemiological features to those identified in prior investigations.	N	N	H	N
[29]	2020	This study was the first comprehensive evaluation that aimed to assess the clinicopathological characteristics of PIOSCC and potential etiological factors related to its prognosis.	N	N	H	H
[30]	2019	According to the results of this study, an OPMD may serve as a risk factor for the development of OC. Although there are active clinical trials and recommendations to remove high-risk lesions, there are currently no effective chemopreventive strategies available.	N	N	M	H
[31]	2021	The findings of this study implied that cutting-edge AI methods can contribute in an unobtrusive way to the early detection of OC.	H	H	M	M
[32]	2003	According to this research, ameloblastoma cases should be thoroughly examined to discover small histological changes that could indicate aggressive behavior by comparing the tumors’ histologic pattern to their biological behavior.	N	N	L	L
[33]	2022	This study’s conclusions demonstrated the need for all patients with non-healing lip lesions to have a full physical examination that includes an intraoral examination and a review of their medical history. When lip cancer is accurately diagnosed and staged, it can be treated quickly and with the best surgical procedure possible for the greatest results.	N	N	L	N
[17]	2022	This research demonstrated the value of ML applications for the prognosis and treatment of potentially malignant (pre-cancerous) oral lesions.	H	M	L	L
[18]	2022	ML and DL classification techniques for OC detection were studied in this study. Several studies revealed that the ML model works admirably in diagnostic and prognostic investigations of oral cancer. To be used in routine clinical practice, these models need to be enhanced to increase their interpretability and they require external evaluation for generalizability utilizing deep hybrid learning approaches.	H	H	L	L
[19]	2021	Most OC outcomes can be predicted using ML algorithms with good accuracy. Furthermore, this study concluded that because these outcomes are uncommon, it is necessary to use class imbalance strategies to handle the skewness of the data.	H	H	L	L

H—High-level discussion, M—Moderate-level discussion, L—Low-level discussion, N—Not available.

**Table 3:** A list of articles on ML models for oral cancer diagnosis.

Ref.	ML Approaches Used	Data Set	Computation Tools	Features Extracted/Features Selected	Feature Extraction Approach	Key Contribution	Limitations	Performance Evaluation Metrics
[144]	LR, DT, SVM, and K-NN	467 OSCC patients	MATLAB R2020a	Prognostic features	PCA and bivariate analysis	It will allow the clinicians to predict the progression of the disease.	Genetic profiling, biomarker analysis, and sophisticated histopathology imaging were missing from this paper.	Acc = 0.705, specificity = 0.841, sensitivity = 0.41
[163]	SVM, GMM	The 1194 cells were taken from 341 healthy and 429 OSF with dysplasia photos.	Snake tool for image segmentation	Hyperchromasia, and nuclear texture, 23 characteristics were derived from segmented biopsy pictures.	Active contour method of gradient vector flow (GVF).	A median filtering technique is suggested for image pre-processing to get rid of the noise.	Expert’s topic expertise and the right image processing were absent.	Acc = 99.66%
[164]	CNN, Gabor filter, Random forests	High-grade = 15 Low-grade = 25 and Healthy = 2 subjects.	Computer aided automatic tools	Texture-based features	Gabor feature extraction	The identification of keratin pearls and the segmentation of subepithelial and epithelial layers can be used for oral precancerous screening and OSCC grading, respectively.	Very little research was carried out on cytopathological and histological pictures to identify the keratin pearl structure.	Acc = 99.88%
[165]	ANN	211 cases with OSCC were identified between 1990 and 2000.	Statistical tools	Age and gender of the patient during the time of diagnosis were considered when data were analyzed.	Peri-tumoral inflammatory infiltrate with local recurrence	This study’s goal was to ascertain whether patients with OSCC may have their 5-year survival rate and incidence rate of LR affected by the presence and grade of PTI	It was not possible to determine involvement in other age-related cancers.	Specificity = 90.59%, sensitivity = 67.74%, Acc = 78.56%
[166]	LR, linear SVM	A total of 34 patients were enlisted for tissue biopsies of suspected oral epithelial lesions.	-	Spectral, time-resolved, and autofluorescence features.	Linear discriminant analysis	They created a CAD system that used ML to automatically distinguish between malignant and healthy oral tissue using data from in vivo widefield maFLIM endoscopy.	In this study, numerous spectra per individual were used as separate datasets, resulting in training and testing sets that were not genuinely independent.	F1 score = 0.85, specificity = 74%, sensitivity = 94%
[131]	SVM, RF, LR, and K-NN	High-definition cytology photos	Telectology platform	Mitotic figures, hyperchromatic nucleus, multiple nuclei, etc.	Field of view extraction method	This study thus prove the value of tele cytology for accurate, remote diagnosis and the application of autonomous ANN-based assessment to increase its efficiency.	According to the limitations of traditional cytology, OPML can only be recognized with a poor sensitivity of approximately 18%.	It demonstrated an accuracy result of 84 to 86% in the identification of oral lesions
[167]	LR, RF, SVM, NB	145 patients suffering from early stage OTSCC.	GridSearchCV, StratifiedKFold, and sklearn Python tools.	Simple clinical and pathologic characteristics linked to patients’ prognoses were the factors used for this investigation.	-	They proved that the best approach is not to create an application that blends ML algorithms with an EHR system.	Lack of large training sets and samples.	The best results were achieved by the random forest model (specificity = 75%: sensitivity = 85%; AUC = 0.786.
[168]	KNN	Using a cytology-on-a-chip method, 999 patients had OSCC and PMOLs.	Data visualization tools, cytopathology tools	144 cellular/nuclear features were gathered from single-cell analyses.	PCA	The results of the present study demonstrated the benefit of a POC-amenable cytology platform that can detect and monitor oral lesions throughout the full spectrum of OED diagnoses.	The present study was limited by the fact that past investigations of cytology adjuncts and POCOCT, in general, focused primarily on PMOL examination in secondary conditions or clinical settings, where malignant and dysplastic lesions could be significantly more prevalent compared to the primary clinical setting.	Acc = 99.3 %

**Table 4:** A list of articles on DL models for oral cancer diagnosis.

Ref.	DL Approaches Used	Data Set	Computation Tools	Features Extracted/Features Selected	Feature Extraction Approach	Key Contribution	Limitations	Performance Evaluation Metrics
[202]	CNN	160 oral cancer images	Tensorflow	Texture features	Local binary pattern	This paper developed a methodology using the DL algorithm to classify oral images into either normal or abnormal images.	The DL models used in this paper were quite outdated.	Acc = 0.99, specificity = 0.99, sensitivity = 0.98
[203]	DeepSurv, Cox proportional hazard model (CPH), random survival forest (RSF)	255 images	Python packages such as Lasagne, tensorboard_logger, etc.	9 features: T stage, N stage, HG, PNI, ENE, LVP, OR, BM, and RM.	-	This model can be effective in predicting with higher accuracy and can guide clinicians both in choosing treatment options and avoiding unnecessary treatments	Statistical methods such as regression trees and classification might be intuitive for clinicians, but they suffer from poor performance and high variance.	DeepSurv showed the finest performance with Acc = 0.810
[204]	TILAb. ResNet50, DenseNet, Inception-v3, Xception	70 cases, containing 10 control cases and 60 OSCC patients.	Tensor-flow, OpenSlide, Sickit-Learn, Matplotlib, NumP, and Pandas.	Histological and pathological features	Patch-based feature extraction approach	The proposed framework for automated quantification of TILs, computation of their abundance score, and its prognostic analysis of patient survival using OSCC histology images is the first of its kind.	Difficulties in managing OSCC patients include early recurrence, frequent lymph node metastases, and extra nodal extension.	AUC = 0.98
[205]	VGG-16 CNN	170 image pairs	Android studio	Pre-cancerous and cancerous lesions	-	Created low-cost but powerful smartphones are promising developments for the creation of low-cost, portable, simple-to-use autofluorescence imaging devices for oral cancer detection.	It would not work for professionals who are in remote areas.	Acc = 0.94
[206]	3DCNN, 2DCNN	7000 CT images of early oral cancers	Caffe, CT	Topology features such as pixel and audio	The 3DCNN automatically extracts features from the dataset	The results proved that 3DCNN can better identify benign and malignant lesions of early oral cancers	Due to space limitations, this paper only discussed a single sequence of images, without combining different imaging modalities.	3DCNN AUC = 0.801
[207]	CNN, Capsnet	82 malignant and 68 benign slide images were obtained from the GDC portal.	Tensorflow	Visual features	Artisanal feature extraction method	The capsule network is suitable for identifying histopathological images in early stage oral cancer.	CNN is not resilient to significant input data modifications	Sensitivity = 0.9778 Specificity = 0.9692 ACC = 97.35%
[208]	CNN	45 OSCC patients had CT scans of 127 cervical lymph nodes that were verified to be positive and 314 cervical lymph nodes that were discovered to be negative.	DIGITS library was used to implement the AlexNet architecture on the Caffe framework.	Image features	-	This study evaluated the efficacy of DL image categorization for the detection of lymph node metastases	The image segmentation was carried out manually; hence the model did not work in real time.	Acc = 0.782 Sensitivity = 0.754 Specificity = 0.81
[209]	CNN (AlexNet)	Cone-beam CT (CBCT) 3D dental imaging	Veraviewepocs 3D, Alphard VEGA	The test ROIs were classified into seven tooth types by the trained network	It was carried out through convolution and pooling layers.	The proposed method is advantageous in obtaining high classification accuracy without the need for precise tooth segmentation.	The major limitations of this study were the small amount of evaluation data and the independent evaluation of slice images.	Acc = 0.88

**Table 5:** Open Challenges for Oral Cancer Diagnosis.

References	Difficulty in Getting Accuracy	Choosing Correct Features	Data Privacy and Confidentiality	Challenges in Deep Learning/Machine Learning	Trustworthy AI
[36]	✓	✓	✓	✓	✓
[131]	×	✓	×	×	✓
[134]	×	✓	✓	×	✓
[139]	✓	✓	×	✓	✓
[209]	✓	✓	×	✓	✓
[225]	✓	✓	✓	✓	✓
[226]	✓	✓	×	✓	✓
[227]	✓	✓	✓	✓	✓

## Data Availability

Not applicable.
